# Deep learning classification models demonstrate high accuracy and clinical potential in radiograph interpretation in the arthroplasty clinical pathway: A systematic review and meta‐analysis

**DOI:** 10.1002/jeo2.70342

**Published:** 2025-07-13

**Authors:** Omar Musbahi, Amal Ahmed, Thomas Hall, Kamila Kutyla, Muhayman Sadiq, Justin Cobb, Alex Bottle, Richard Van Arkel, Gareth Jones

**Affiliations:** ^1^ Imperial College London, MSk Lab London UK; ^2^ Imperial College London School of Public Health London UK; ^3^ Department of Mechanical Engineering Imperial College London London UK

**Keywords:** arthroplasty, artificial intelligence, convolutional neural network, deep learning, radiology

## Abstract

**Purpose:**

Imaging is a cornerstone of the osteoarthritis (OA)‐arthroplasty clinical pathway: the continuum of care that patients with OA undergo, from initial diagnosis through to arthroplasty and postoperative follow‐up. With growing interest from the orthopaedic community, this meta‐analysis broadly evaluates the performance of deep learning algorithms in interpreting radiographs and cross‐sectional imaging in this pathway. The authors hypothesise that deep learning algorithms will have comparable performance to clinicians when interpreting radiographs, but not cross‐sectional imaging, with negligible difference in diagnostic and prognostic tasks.

**Methods:**

Ovid Medline, Ovid Embase, Scopus and Web of Science were searched for studies published between January 1, 2012, and April 1, 2024, evaluating deep learning algorithms for diagnostic and prognostic tasks along the pathway. Eligible studies included those that used established diagnostic or surgical candidacy assessments as ground truth. Study quality was assessed using the Quality Assessment of Diagnostic Accuracy Studies 2 tool, and pooled sensitivity and specificity were determined. Hierarchical summary receiver operating characteristic curves assessed diagnostic performance.

**Results:**

The meta‐analysis of artificial intelligence (AI) interpretation included 66 studies, with a pooled sensitivity of 0.88 (95% confidence interval [CI]: 0.81–0.92) and a pooled specificity of 0.91 (95% CI: 0.87–0.94). Sensitivity and specificity values were higher for AI interpretation of radiographs (55 studies) compared to cross‐sectional imaging, with no significant difference in performance between diagnostic and prognostic tasks. For clinician interpretation, 11 studies showed a pooled sensitivity of 0.76 (95% CI: 0.64–0.85) and a pooled specificity of 0.79 (95% CI: 0.59–0.90).

**Conclusions:**

This meta‐analysis highlights the potential of deep learning algorithms to improve efficiency in OA classification and prognosis in the arthroplasty pathway based on low‐to‐moderate quality evidence. Although the results are not generalisable, the findings suggest deep learning models have the potential to be adopted in OA treatment pathways, warranting further exploration of its role in patient care.

**Level of Evidence:**

Level III.

**Abbreviations**
AI: artificial intelligence
AUC: area under curve
CNN: convolutional neural network
CT: computed tomography
DenseNet: dense convolutional network
DL: deep learning
GAN: generative adversarial network
GMLP: good machine learning practice
HSROC: hierarchical summary receiver operating curve
LSTM: long short‐term memory
MRI: magnetic resonance imaging
OA: osteoarthritis
PET: positron emission tomography
PICO: patient/population, intervention, comparison and outcomes
PRISMA: preferred reporting items for systematic reviews and meta‐analyses
QUADAS‐2: quality assessment of diagnostic accuracy studies 2
ResNet: residual network
RNN: recurrent neural network
SPECT: single photon emission computed tomography
THA: total hip arthroplasty
VGG: visual geometry group
ViT: vision transformers

## INTRODUCTION

Osteoarthritis (OA) is a condition that affects over 500 million people worldwide, with over 40 million diagnosed in 2019 according to the latest Global Burden for Disease study [119]. End‐stage treatment of OA typically requires arthroplasty, and, like the incidence of OA, the number of patients undergoing arthroplasty is also increasing. In 2019, over 450,000 total knee arthroplasties and more than 260,000 total hip arthroplasties were performed in the United States, accounting for over 2% of adults aged 50 and older [95, 112, 148]. From the initial presentation of symptomatic OA to postoperative assessment after arthroplasty, imaging is a key pillar of the OA clinical pathway [30, 57].

Amidst the increasing incidence of OA and demand for arthroplasty, there has been a concomitant rise in imaging volumes—a cornerstone of the OA‐arthroplasty clinical pathway. Automated image analysis is emerging as a potential solution for improving efficiency, accuracy and consistency in imaging interpretation across various stages of the pathway [55, 87]. Artificial intelligence (AI) is being deployed in medical image analysis in a variety of clinical settings, showing the potential to serve as a useful and efficient adjunct to human‐only interpretation [78]. In the musculoskeletal field, several authors have demonstrated the advantages of AI‐based image analysis for many types of tasks, including aiding diagnosis and prognosis [22, 69, 74]. In the OA‐arthroplasty clinical pathway, AI has been used for disease severity grading [23, 85], alignment measurement [141, 150], and the detection of postoperative complications [155]. However, despite increasing research into the integration of AI in orthopaedic imaging, to date, there has not been a systematic review that overviews the utility of AI for diagnostic and prognostic purposes on radiographs and cross‐sectional imaging in the arthroplasty pathway. Understanding the utility of AI for these purposes across different imaging modalities is important for guiding for future research and appreciating where AI can best be implemented into clinical practice in the future.

Amidst strong interest from the orthopaedic community and the first wave of regulatory approvals [35], the current evidence for AI‐based image analysis in the OA‐arthroplasty clinical pathway was quantitatively appraised. This study aimed to evaluate the efficacy of AI‐based algorithms in interpreting radiographs and cross‐sectional imaging for diagnostic and prognostic purposes across all stages of the clinical pathway and compare their performance to that of human clinicians. Focusing on deep learning, a class of AI well‐suited to image analysis, a broad systematic review summarising the performance of AI across all stages of the clinical pathway was performed. The accuracy and associated biases of the algorithms in the dataset were assessed in the meta‐analysis. Furthermore, the efficacy of AI in interpreting radiographs and cross‐sectional imaging for diagnostic and prognostic purposes was compared. A comparison between the overall performance of AI‐based algorithms and human clinician performance was undertaken. The authors hypothesise that deep learning algorithms will show comparable performance to human clinicians when interpreting imaging for both diagnostic and prognostic purposes on radiographs, but significantly worse performance on cross‐sectional imaging in comparison.

## METHODS

This study was performed in accordance with the preferred reporting items for systematic review and meta‐analysis (PRISMA) guidelines and registered prospectively on PROSPERO CRD42020198878.

### Search strategy and selection criteria

In this study, the OA‐arthroplasty clinical pathway was defined as the care pathway for patients with OA spanning from initial diagnosis and classification to postoperative care and follow‐up. Studies applying deep learning to medical imaging at any stage of the OA‐arthroplasty clinical pathway, including the evaluation of preoperative conditions (e.g., OA) and postoperative complications (e.g., aseptic loosening), were searched for. All major joints (hip, knee, ankle, elbow and shoulder) and the most common imaging modalities were considered: plain radiography (X‐ray), computed tomography (CT), magnetic resonance imaging (MRI), single‐photon emission computed tomography (SPECT), position emission tomography (PET) and scintigraphy.

The use of deep learning for image interpretation was considered to be the intervention within the context of the population, intervention, comparison and outcome (PICO) framework. For the purposes of comparability between studies, only classification algorithms were considered. Contextual examples of classification tasks include diagnosing disease, grading its severity, predicting its progression or outcome, detecting postoperative complications and identifying implant models. Acquisition, preprocessing and segmentation were deemed out of scope as they are evaluated using alternative or inconsistent metrics, and are conceptually different.

The search covered the following databases: Ovid‐MEDLINE, Embase, Scopus and Web of Science, with full search terms available as File S1. Where appropriate, search terms were mapped to either medical subject headings (MeSH) or non‐MeSH. Further articles were also identified by manually searching the bibliographies, citations and related articles (as recommended by PubMed) of included articles. For contemporaneity, the search only considered studies published between 1 January 2012 and 1 April 2024. The earlier date corresponded to the publication year of AlexNet, which is widely considered a paradigm shift in the performance of Deep Learning algorithms in image classification tasks [67]. Only original research articles were considered eligible for the systematic review, inclusive of conference proceedings, letters, preprints and scientific reports. The search was addressed to English articles, but no articles were discounted due to their language.

### Eligibility assessment

Articles were first screened by two independent reviewers based on titles and abstracts (A.A. and K.K.); nonconsensus was resolved by a third reviewer (O.M.). The full text of candidate articles was reviewed before the final selection. Where the same content formed substantially part of two separate publications by the same authors, only the most developed study was included, for example, the study with the greatest number of subjects. Studies by the same authors but with a substantive change, such as applying the same framework to a different database, were deemed to be distinct and both were included.

Nonhuman and ex vivo studies were excluded. However, no other limitations were placed on the demographics of each included study. Age and sex were not reported given the nature of the study. Only pure approaches to image interpretation using deep learning were considered eligible; hybrid methods that required clinician input to prescore or segment images were excluded. For studies where clinician interpretation results were collected, these interpretations could have come from radiologists, orthopaedic surgeons or both.

### Data extraction

The data extracted from selected articles included identifying information such as the title of the study, authors, study type, publication type and digital object identifiers. The study characteristics were also extracted: target anatomy, target condition, purpose, imaging modality, image source, sample size, sample composition (e.g., split between positive‐negative indication), train‐validation‐test split ratios, AI algorithm (neural network architecture), method of acquiring and definition of ground truth and the validation method used.

Performance metrics of the deep learning algorithm and, where possible, comparative performance of the clinician were also investigated. Metrics included specificity, sensitivity, area‐under‐the‐curve (AUC), precision, average precision, F1 score (combines precision and recall scores of a deep learning model to measure its accuracy), Jaccard Index (measures similarity between two sample sets by calculating the ratio of their intersection [similar elements between sets] to their union [the combined size of both sample sets]), Dice coefficient (measures similarity between two sample sets by doubling their intersection term relative to the combined sample size, typically yielding higher values), true positive rate, false positive rate, false negative rate and true negative rate [45, 50]. In this context, sensitivity refers to the performance of AI or clinicians to correctly identify abnormalities on imaging, and specificity refers to the performance of AI or clinicians to avoid false positive diagnoses when interpreting imaging. The Jaccard Index and Dice Score are used to understand similarities between two sample sets: The Jaccard Index is the size of the intersection divided by the size of the union between two sets, and the Dice Score in most cases, these data were extracted from direct reporting in the article. However, in cases where such data was not explicitly stated, it was ascertained—where possible—from other published information, such as by calculating from confusion matrices or other reported performance metrics. Where there was insufficient information, corresponding authors were contacted to supply the required data. Failing this, the study was excluded from further analysis.

### Analysis

A hierarchical summary receiver operating characteristic (HSROC) curve was constructed on STATA using a bivariate random‐effects meta‐analytic model to pool sensitivity and specificity data from individual studies [105]. This approach accounts for the inherent correlation between sensitivity and specificity and incorporates between‐study heterogeneity. The HSROC model estimates key parameters, including accuracy (Λ), threshold (Θ) and asymmetry (β), to characterise the trade‐off between sensitivity and specificity across studies. Pooled sensitivity and specificity were derived from the model's fixed and random effects, with studies weighted by their variance (precision). The AUC, reflecting overall diagnostic performance, was computed directly from the HSROC curve, integrating the relationship between sensitivity and specificity across thresholds. This method ensures robust estimates that are less biased by study heterogeneity or imbalances in sample size. HSROC curves from the study results from the following groups were plotted: overall AI performance, AI performance for diagnostic purposes, AI performance for prognostic purposes, AI performance with radiographs, AI performance with cross‐sectional imaging, and overall human performance. Using study characteristics such as sample size, image source, algorithm choice, train‐test split ratios and ground truth acquisition, a qualitative analysis of best practices was undertaken.

For studies reporting multi‐class outputs, the performance metrics were aggregated into a single representative variable, with the method dependent on the output type: semi‐continuous or discrete. For semicontinuous (scaled) outputs, the outputs were split between positive and negative indications, with performance metrics recalculated. Using the Kellgren–Lawrence classification of OA as an exemplary case, scores ≤2 indicate no, doubtful, or mild evidence of the condition, hence they were aggregated as negative indications. Scores ≥3 describe moderate and severe conditions, and hence were aggregated as positive indications. For discrete (categorical) outputs, such as implant models, the numbers of true positives, true negatives, false positives and false negatives were summed across the respective classes.

Where multiple results were reported within the same research article, such as detecting joint space narrowing, osteophytes and subchondral cysts separately, the best‐ and worst‐performing cases were included in the aggregated analysis as separate data points. For subset analyses, individual results were included where appropriate.

The QUADAS‐2 framework, a tool used to assess the quality of diagnostic accuracy studies in systematic reviews, was used to evaluate the risk of bias in each study. The framework evaluates studies across four domains: patient selection, index test, reference standard and flow and timing, focusing on the risk of bias and applicability concerns [149].

## RESULTS

### Search results

**Figure 1:**
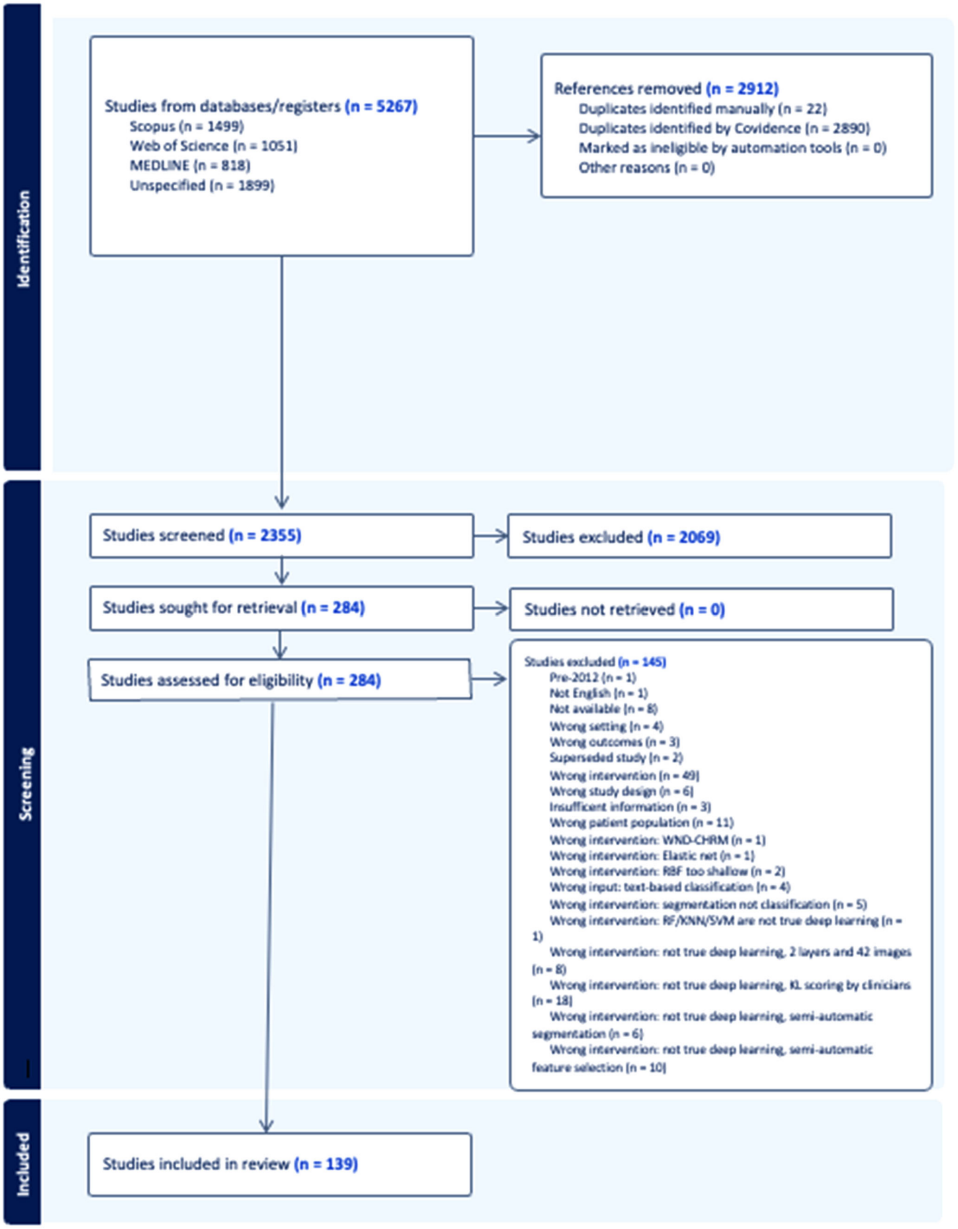
Preferred reporting items for systematic reviews and meta‐analysis flow diagram.

5627 peer‐reviewed studies were identified, of which 2912 were duplicates. Figure 1 highlights the PRISMA flow diagram. One hundred and thirty‐nine studies were included in the systematic review after full‐text screening [1, 2, 3, 4, 5, 6, 7, 8, 9, 10, 11, 12, 13, 14, 15, 16, 17, 18, 19, 20, 21, 23, 24, 25, 26, 27, 28, 29, 31, 32, 33, 34, 36, 37, 38, 39, 40, 41, 42, 43, 44, 46, 47, 48, 49, 51, 52, 53, 54, 56, 58, 59, 61, 62, 64, 65, 66, 68, 70, 71, 72, 73, 75, 76, 77, 79, 80, 81, 82, 83, 86, 88, 89, 90, 91, 92, 93, 94, 96, 97, 98, 99, 100, 101, 102, 103, 104, 106, 107, 108, 109, 110, 111, 113, 114, 115, 116, 117, 118, 120, 121, 122, 123, 124, 125, 126, 127, 128, 129, 130, 131, 132, 133, 134, 135, 136, 137, 138, 139, 140, 142, 143, 144, 145, 146, 147, 151, 152, 154, 156, 157, 158, 159, 160, 161, 162, 163, 164, 165] (30 studies from cross‐referencing) and 68 (43%) met the criteria for meta‐analysis. Radiography (*n* = 121; 88%) and MRI (*n* = 18; 13%) were the primary imaging modalities. Almost all studies deployed convolutional neural networks (CNNs), most commonly off‐the‐shelf architectures, such as DenseNet (dense convolutional network), ResNet (residual network), EfficientNet, Inception, Inception‐ResNet and VGG (visual geometry group) [153, 166]. Most studies used deep learning for disease classification or diagnosis (*n* = 105; 76%), followed by preoperative planning (*n* = 12; 8%) and progression prediction or prognosis (*n* = 11; 8%). The most common application was grading the severity of OA, with 85 studies (62%) targeting Kellgren–Lawrence gradings.

### Quality of assessments

The quality assessment of the included studies using the QUADAS‐2 tool revealed variability in the risk of bias across domains. For patient selection, 40% of studies were rated as ‘low risk’, and 35% were rated as ‘high risk’. In the reference standard domain, 10% were rated as ‘high risk’. Overall, across all domains, 57% of studies had a ‘low risk’ of bias, and 17% were rated as ‘high risk’. These results highlight variability in methodological rigor, particularly in the patient selection domain, which showed the greatest proportion of high‐risk studies.

### Pooled analysis

**Figure 2:**
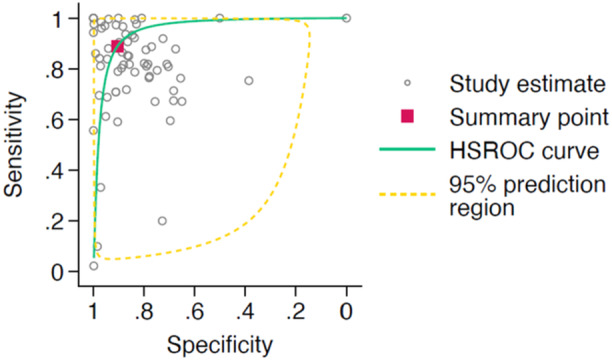
Hierarchical summary receiver operator characteristic (HSROC) curve of study estimated sensitivity plotted against specificity for deep‐learning only interpretations.

**Figure 3:**
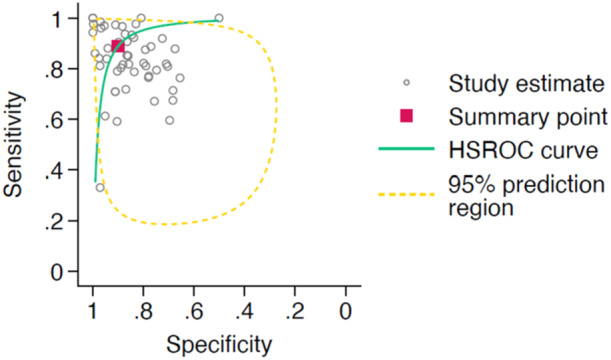
Hierarchical summary receiver operator characteristic (HSROC) curve of study estimated sensitivity plotted against specificity for deep‐learning only interpretations for diagnostic purposes.

**Figure 4:**
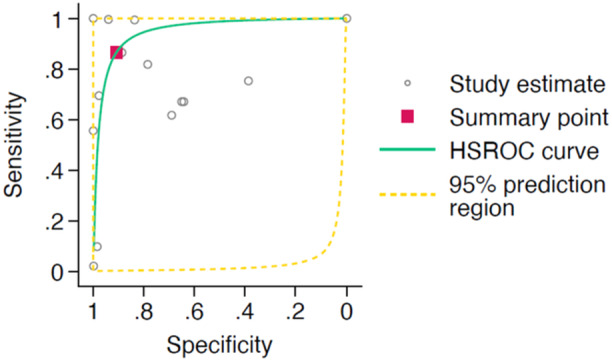
Hierarchical summary receiver operator characteristic (HSROC) curve of study estimated sensitivity plotted against specificity for deep‐learning only interpretations for prognostic purposes.

A total of 66 studies met the criteria for meta‐analysis of AI‐interpretation performance, with the corresponding HSROC curve shown in Figure 2. The studies had a pooled sensitivity of 0.88 (95% CI: 0.81–0.92) and a pooled specificity of 0.91 (95% CI: 0.87–0.94). When assessing AI interpretation performance for diagnostic purposes, the 52 studies had a pooled sensitivity of 0.89 (95% CI: 0.84–0.93) and a pooled specificity of 0.90 (95% CI: 0.86–0.93). The 12 studies assessing AI interpretation performance for prognostic purposes showed a pooled sensitivity of 0.81 (95% CI: 0.43–0.96) and a pooled specificity of 0.93 (95% CI: 0.79–0.98). The HSROC curves for AI interpretation for diagnostic and prognostic purposes is shown in Figures 3 and 4 respectively.

**Figure 5:**
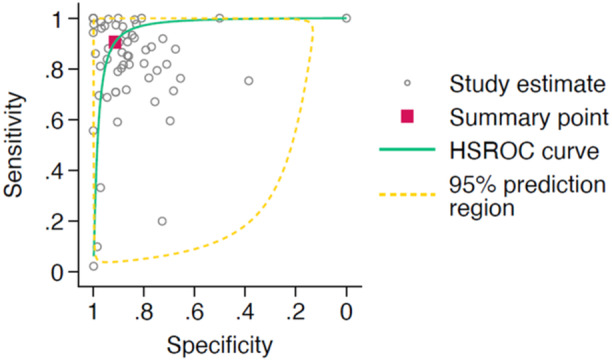
Hierarchical summary receiver operator characteristic (HSROC) curve of study estimated sensitivity plotted against specificity for deep‐learning only interpretations of radiographs.

**Figure 6:**
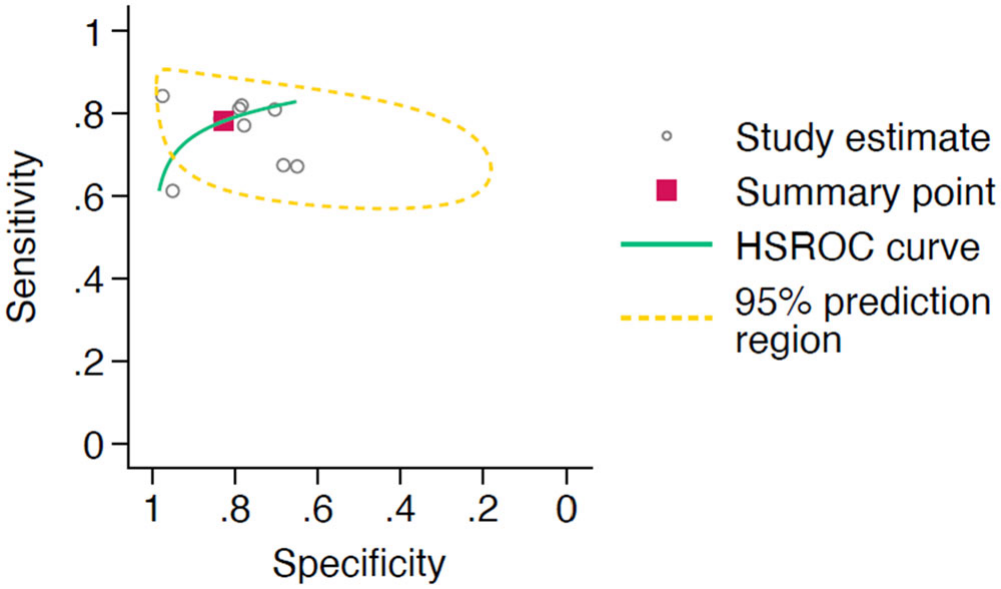
Hierarchical summary receiver operator characteristic (HSROC) curve of study estimated sensitivity plotted against specificity for deep‐learning only interpretations of cross‐sectional imaging.

A total of 55 studies investigated the performance of AI interpretation of radiographs (for both diagnostic and prognostic purposes), with the HSROC curve for these studies shown in Figure 5. These studies had a pooled sensitivity of 0.89 (95% CI: 0.83–0.94) and a pooled specificity of 0.92 (95% CI: 0.88–0.95). Eleven studies investigated the performance of AI interpretation of cross‐sectional imaging (for both diagnostic and prognostic purposes), 10 of which specifically looked at MRI interpretation. The studies had a pooled sensitivity of 0.78 (95% CI: 0.74–0.81) and a pooled specificity of 0.83 (95% CI: 0.72–0.90), with the corresponding HSROC shown in Figure 6.

**Figure 7:**
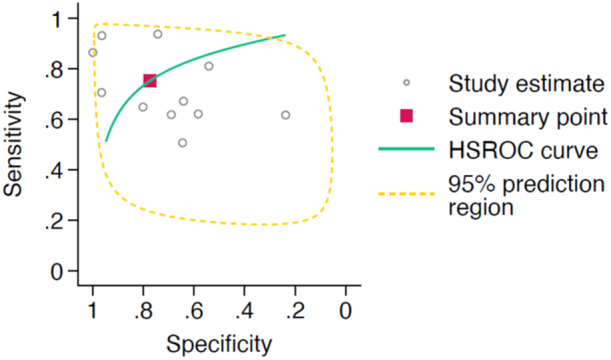
Hierarchical summary receiver operator characteristic (HSROC) curve of study estimated sensitivity plotted against specificity for clinician‐only interpretations.

The HSROC curve for the 11 studies that meet the criteria for meta‐analysis of clinician‐only interpretation performance is shown in Figure 7. Clinicians had a pooled sensitivity of 0.76 (95% CI: 0.64–0.85) and a pooled specificity of 0.79 (95% CI: 0.59–0.90).

**Figure 8:**
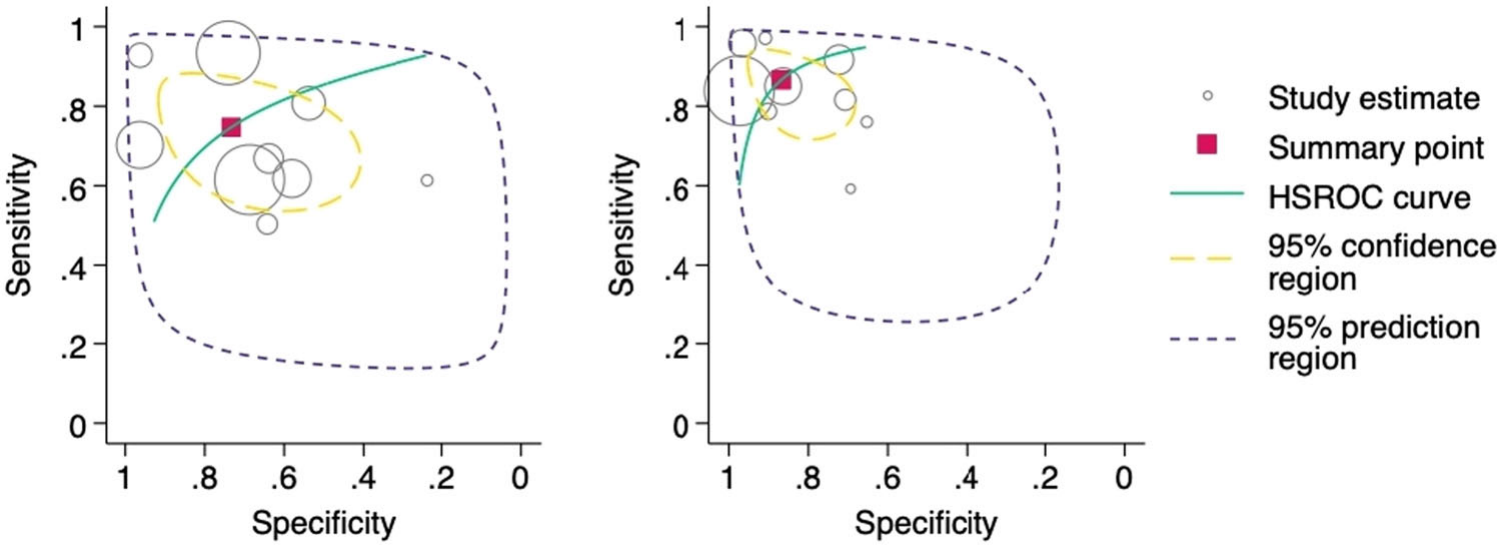
Hierarchical summary receiver operator characteristic (HSROC) curves of study estimated sensitivity plotted against specificity for the nine studies directly comparing artificial intelligence (figure on right) and clinician interpretations (figure on left).

In the meta‐analysis of diagnostic accuracy, nine studies that made a direct comparison between AI performance and human clinicians alone were included. Among these ‘matched’ studies, the pooled sensitivity for AI was 0.87 (95% CI: 0.78–0.92), and the pooled specificity was 0.87 (95% CI: 0.76–0.93). In comparison, human clinicians achieved a pooled sensitivity of 0.75 (95% CI: 0.62–0.84) and a pooled specificity of 0.73 (95% CI: 0.53–0.87). The HSROC curve for these studies is shown in Figure 8.

## DISCUSSION

A high pooled sensitivity and pooled specificity for AI interpretation were revealed in the meta‐analysis, albeit with considerable overlap in sensitivity and specificity results for human interpretation. There was also significant overlap in pooled sensitivity and specificity results of AI results for both diagnostic and prognostic purposes. However, the included models showed higher pooled sensitivity and specificity values for AI interpretation of radiographs compared to cross‐sectional imaging, with little‐to‐no overlap in sensitivity and specificity results.

This study's results are consistent with previous reviews evaluating deep learning in specific imaging tasks. Mohammadi et al. previously compared machine learning/AI with clinicians in OA detection across 27 studies, demonstrating a pooled sensitivity and specificity of 0.88 (95% CI: 0.86–0.91) and 0.81 (95% CI: 0.75–0.85) respectively [84]. In a scoping review of 12 studies, Gurung et al. also reported that AI‐based tools diagnosed postoperative complications for total hip arthroplasty (THA) with an accuracy of 88.30% [44].

Unlike these two studies, which factored in all aspects of AI for single tasks, this study focused purely on deep learning classification (single task) algorithms and excluded hybrid models to minimise the potential for interpretive bias, thereby allowing for a more objective assessment of the diagnostic performance of deep learning in image interpretation. Despite this, there is variance in the sample size, patient demographics, standardisation of image acquisition, and specific deep learning algorithm being tested across each of the included studies, limiting the generalisability of the results.

A critical element in the evaluation of diagnostic accuracy is the choice of the gold standard against which performance is measured. In this meta‐analysis, studies that used established diagnostic criteria, such as the human‐derived Kellgren‐Lawrence grading system, were included, however, these can be associated with significant limitations. Owing to the broad nature of the meta‐analysis, aggregating different deep learning algorithms throughout different stages of the clinical pathway, the large heterogeneity between studies was accepted. Conversely, with the over‐representation of CNNs and OA detection in this dataset, as opposed to models such as vision transformers (ViTs), recurrent neural networks (RNNs), short‐term nemory networks (LSTMs), generative adversarial networks (GANs) and U‐Net variants, caution would be advisable when generalising this study's findings to other architectures and tasks. Several studies in the literature have also highlighted the added value of segmentation and 3D reconstruction (CT, MRI) on DL performance when interpreting imaging. With the exclusion of acquisition, preprocessing and segmentation studies in this analysis, the results are likely skewed. Similarly, there is a skew towards knees, which limits generalisability to the other joints. Therefore, while the study findings broadly support the potential for AI to be adopted as an adjunct to clinical decision‐making in the OA‐arthroplasty clinical pathway, this study pools results from 138 different datasets across 138 different AI models, limiting the generalisability of the study results, and each application must still be properly validated for clinical use. None of the included models have been widely integrated into existing clinical workflow, limiting the current implications of whether these models truly translate into improved clinical decision‐making for orthopaedic surgeons and radiologists [63].

Translating the growing body of AI research into meaningful clinical applications remains a work in progress. Facilitating the adoption of AI in healthcare, 10 guiding principles for good machine learning practice (GMLP) have been jointly developed by the United States, the United Kingdom and Canadian regulators. Similarly, new guidelines for planning and reporting clinical trials involving AI have been released by the SPIRIT‐AI and CONSORT‐AI [77, 81]. While challenges remain, this study's findings suggest that published AI models demonstrate a promising performance for diagnostic and prognostic purposes in the OA‐arthroplasty pathway, warranting further studies to explore its potential for clinical use long term. Studies should handle the challenging ethical issues affiliated with integrating AI into clinical workflows, including confidentiality of patient information and liability in the setting of an incorrect interpretation.

By quantitatively evaluating AI interpretation performance and comparing it to human interpretation, this study provides insight into AI's capacity to improve clinical decision‐making, particularly in grading disease severity, planning preoperative alignment and detecting postoperative complications. The findings underscore the need for ongoing validation and refinement of AI models to ensure generalisability across diverse patient populations and imaging modalities. Ultimately, integrating AI into clinical workflows has the potential to optimise resource utilisation, reduce diagnostic variability and enhance patient outcomes, making it an invaluable adjunct to human expertise in musculoskeletal imaging.

## CONCLUSIONS

This systematic review and meta‐analysis present low‐to‐moderate quality evidence of strong performance of deep learning models in the OA‐arthroplasty pathway.

The included algorithms showed a stronger performance when interpreting radiographs, and considerable overlap in sensitivity and specificity results when comparing diagnostic and prognostic interpretations. AI interpretation showed significant overlap in sensitivity and specificity results with human clinician interpretation, limiting generalisability of the results. The potential of deep learning extends beyond streamlining clinical pathways, with possible applications in refining surgical decision‐making and aiding the detection of postoperative complications. Further validation of specific uses of specific deep learning models on different imaging modalities is required to understand its full impact on clinical practice.

## AUTHOR CONTRIBUTIONS

**Omar Musbahi**: Conceptualisation; literature search and data analysis; drafting and critical revision. **Amal Ahmed**: Literature search and data analysis; drafting and critical revision. **Thomas Hall**: Conceptualisation; literature search and data analysis; drafting and critical revision. **Kamila Kutyla**: Literature search and data analysis; drafting and critical revision. **Muhayman Sadiq**: Literature search and data analysis; drafting and critical revision. **Justin Cobb**: Conceptualisation; drafting and critical revision. **Alex Bottle**: Conceptualisation; drafting and critical revision. **Richard Van Arkel**: Conceptualisation; drafting and critical revision. **Gareth Jones**: Conceptualisation; drafting and critical revision.

## CONFLICT OF INTEREST STATEMENT

The authors declare no conflicts of interest.

## ETHICS STATEMENT

The authors have nothing to report.

## CLINICAL TRIAL REGISTRATION

PROSPERO registration for systematic reviews—CRD42020198878.

## Supporting information

Supplemental File.

## Data Availability

This study analyses the results of 138 scholarly papers sourced from online journals and databases. Articles were accessed from both public domain resources and via institutional access through Imperial College London, the institution at which this review was undertaken. Accessibility of these papers varies according to reader access privileges and different journal policies. For further details, please see the references section of the manuscript.
