# Supplementary material for: Deep learning classification models demonstrate high accuracy and clinical potential in radiograph interpretation in the arthroplasty clinical pathway: A systematic review and meta‐analysis
Source: J Exp Orthop. 2025 Jul 13;12(3):e70342. doi: 10.1002/jeo2.70342 (PMC12255956; doi:10.1002/jeo2.70342)
Supplement: Supplementary file 1 — Supplemental File. [file JEO2-12-e70342-s001.docx]

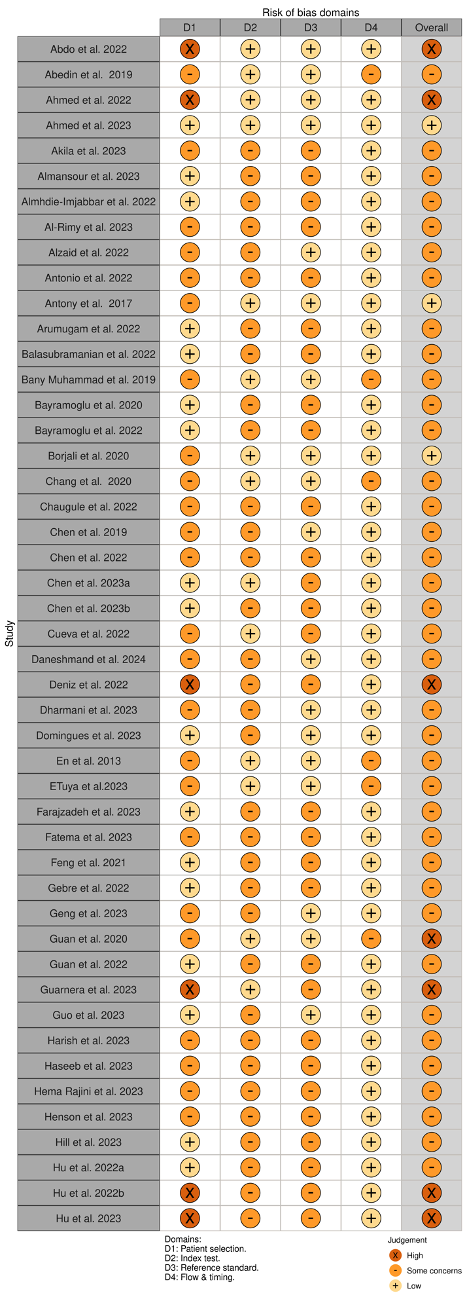


*Methodological quality summary of the included studies using the QUADAS-2 tool*


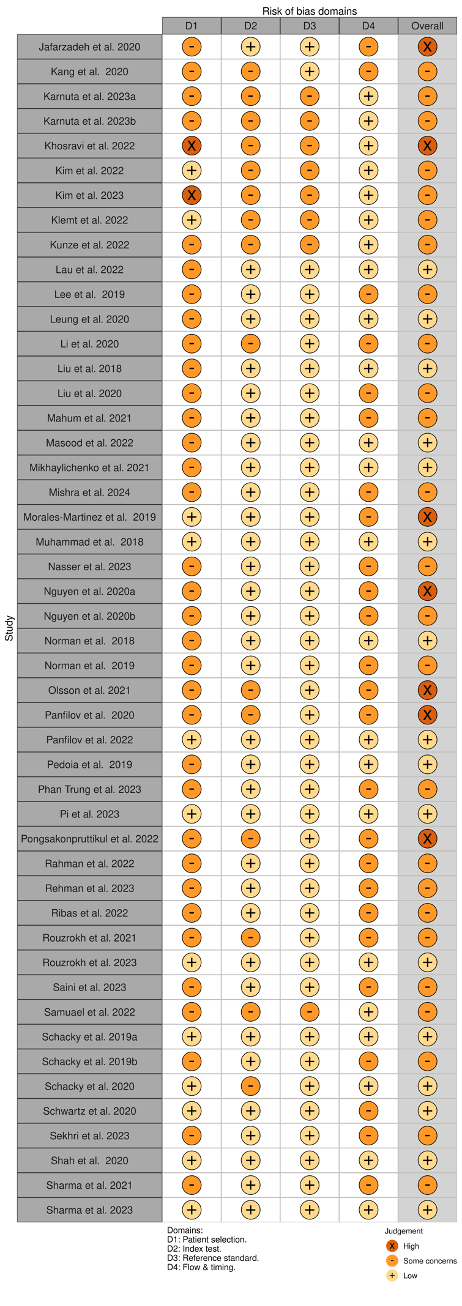
e

*Methodological quality summary of the included studies using the QUADAS-2 tool*


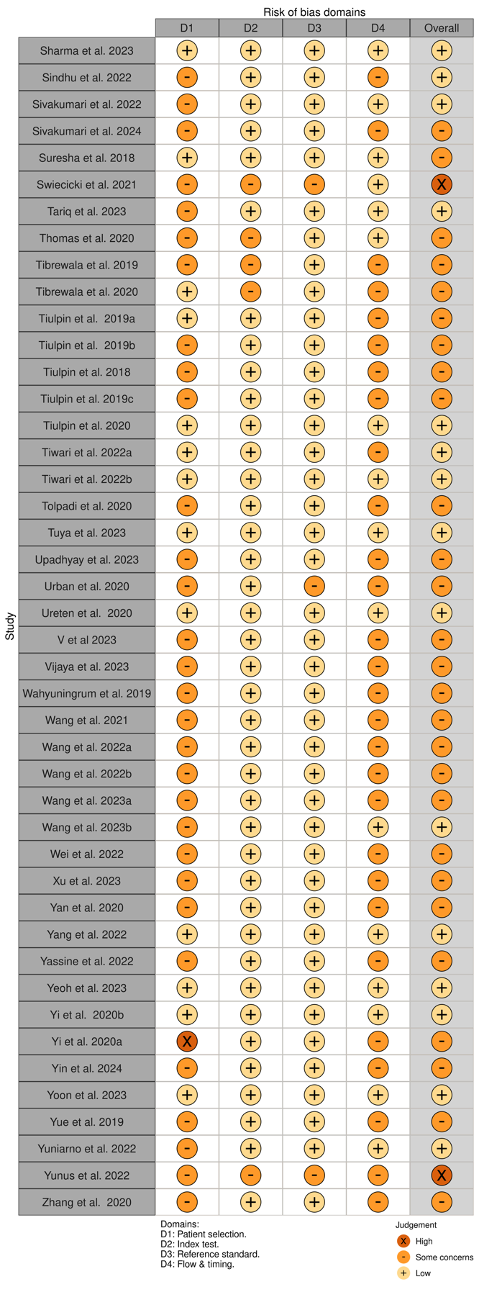


*Methodological quality summary of the included studies using the QUADAS-2 tool*


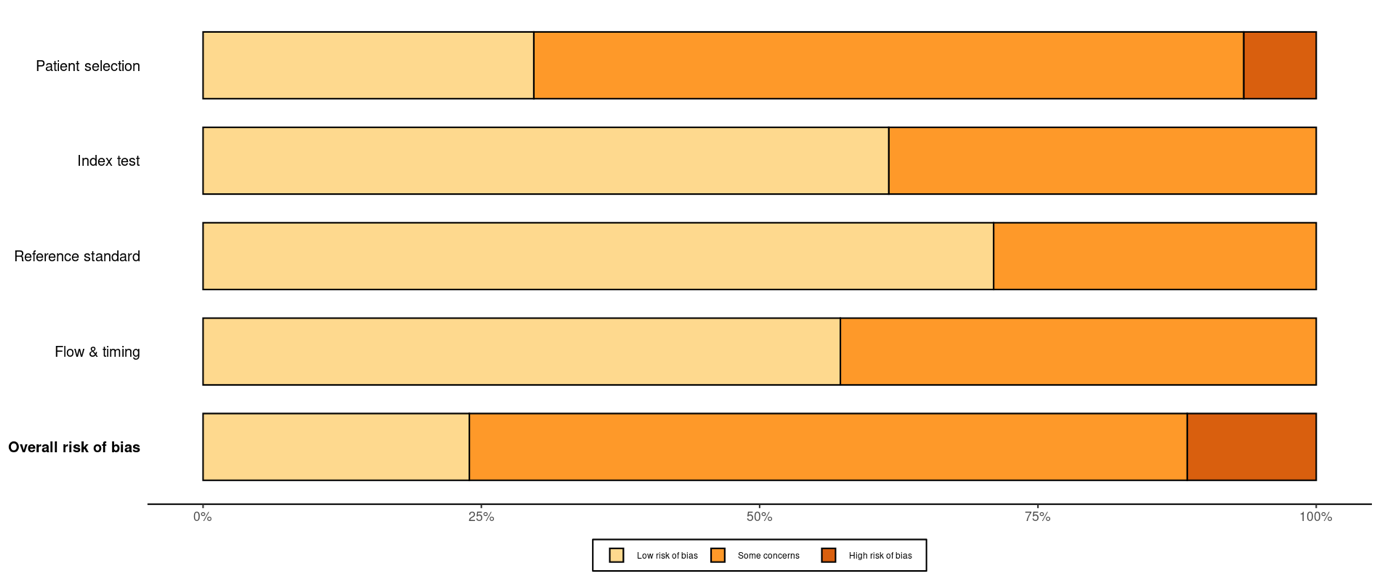


*Methodological quality graph for included studies based on quality assessment utilising QUADAS-2 tool*


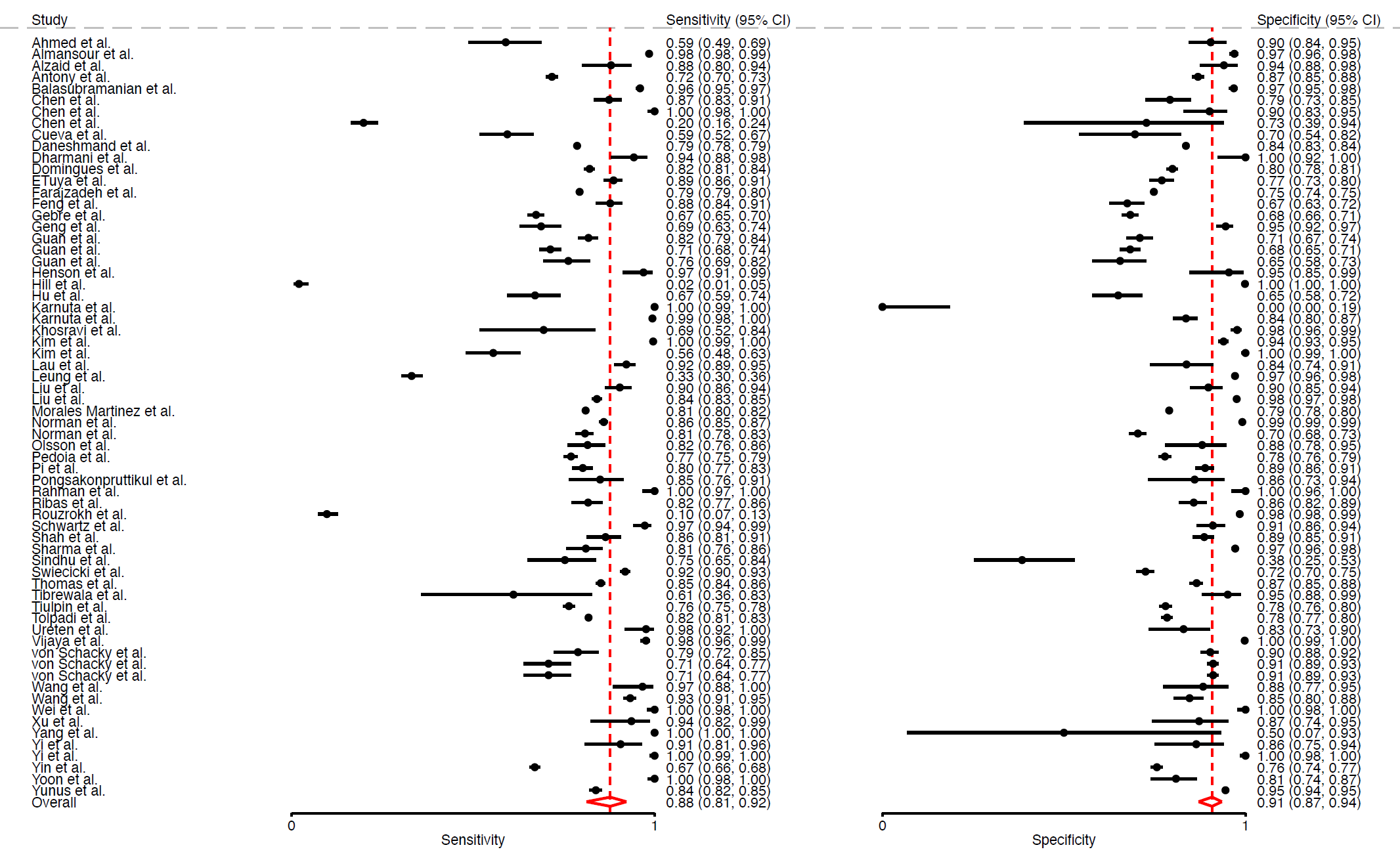
*Meta-Analysis pooled results with estimated sensitivity and specificity values (including 95% confidence intervals) for Deep-Learning only Interpretations overall.*


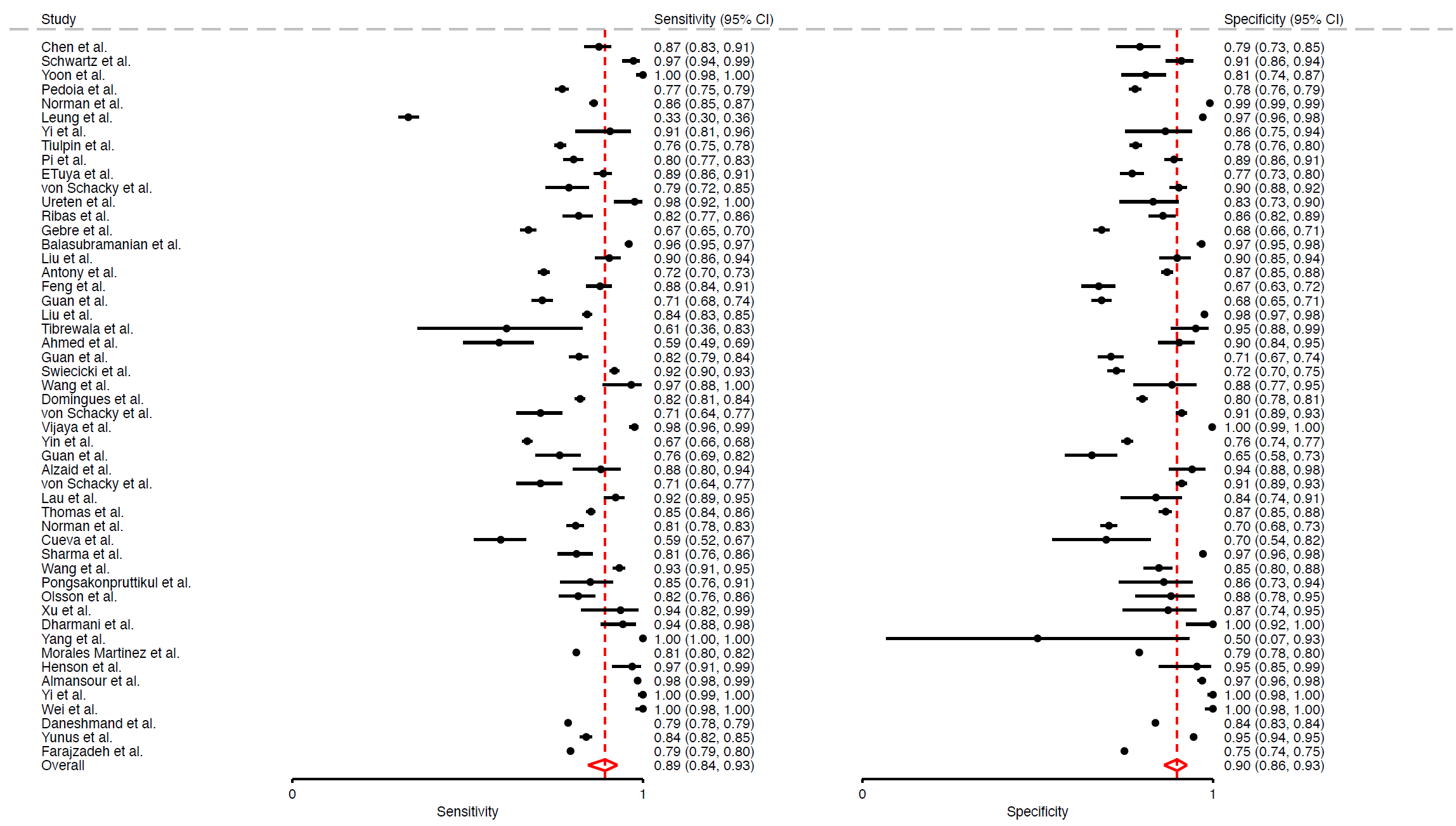


*Meta-Analysis pooled results with estimated sensitivity and specificity values (including 95% confidence intervals) for Deep-Learning only interpretations for diagnostic purposes.*


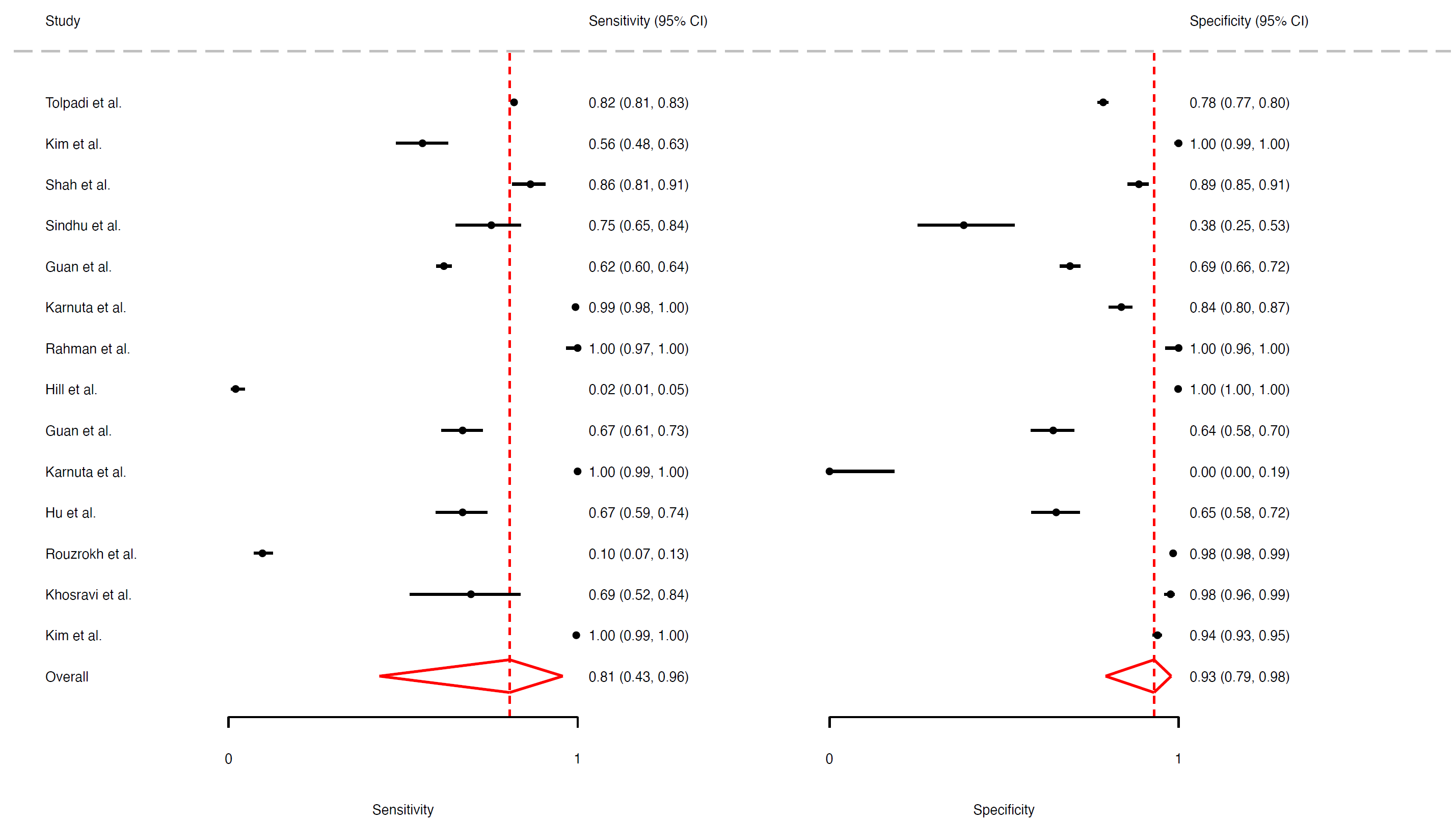


*Meta-Analysis pooled results with estimated sensitivity and specificity values (including 95% confidence intervals) for Deep-Learning only interpretations for prognostic purposes.*


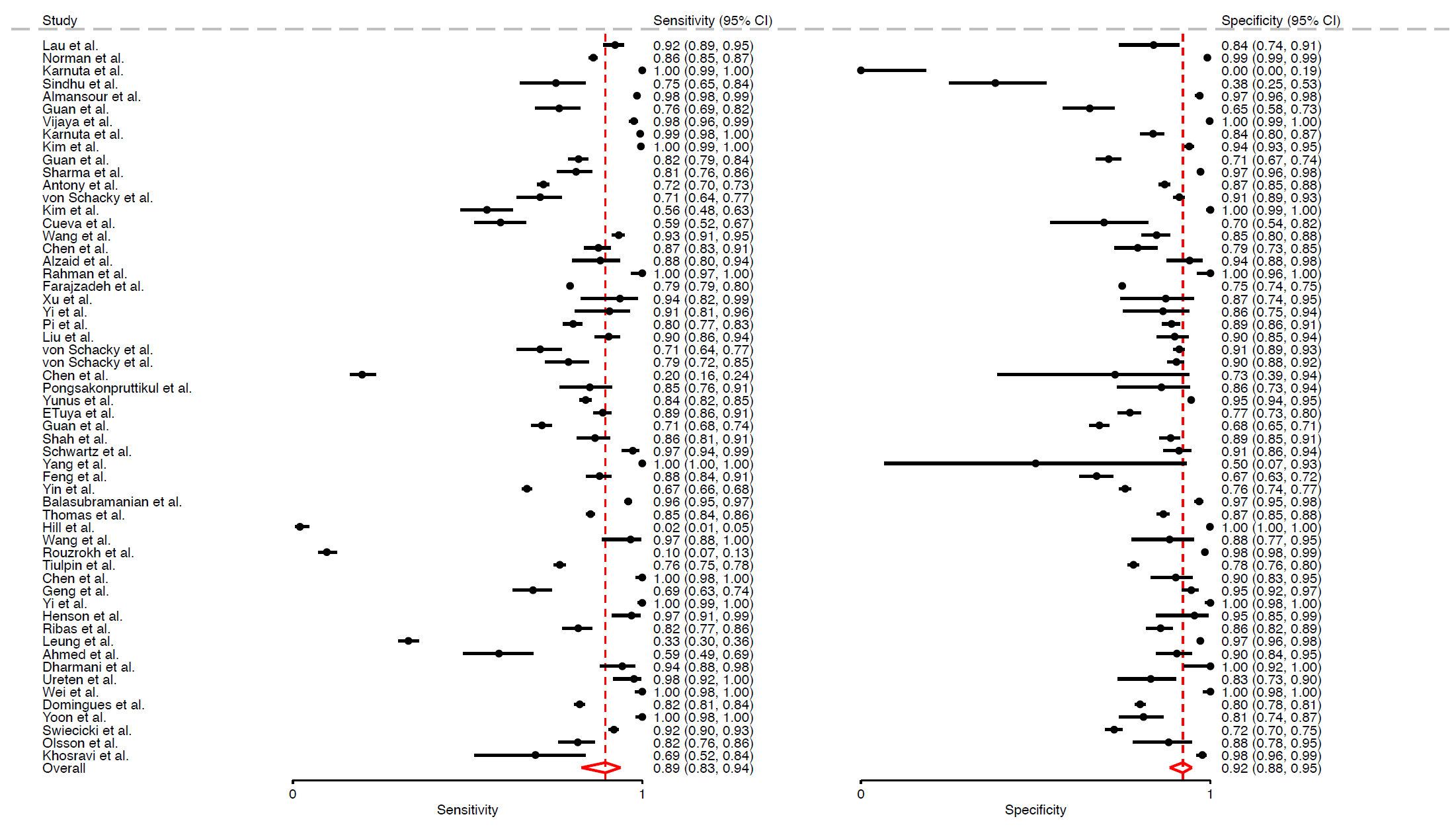


*Meta-Analysis pooled results with estimated sensitivity and specificity values (including 95% confidence intervals) for Deep-Learning only interpretations of radiographs.*


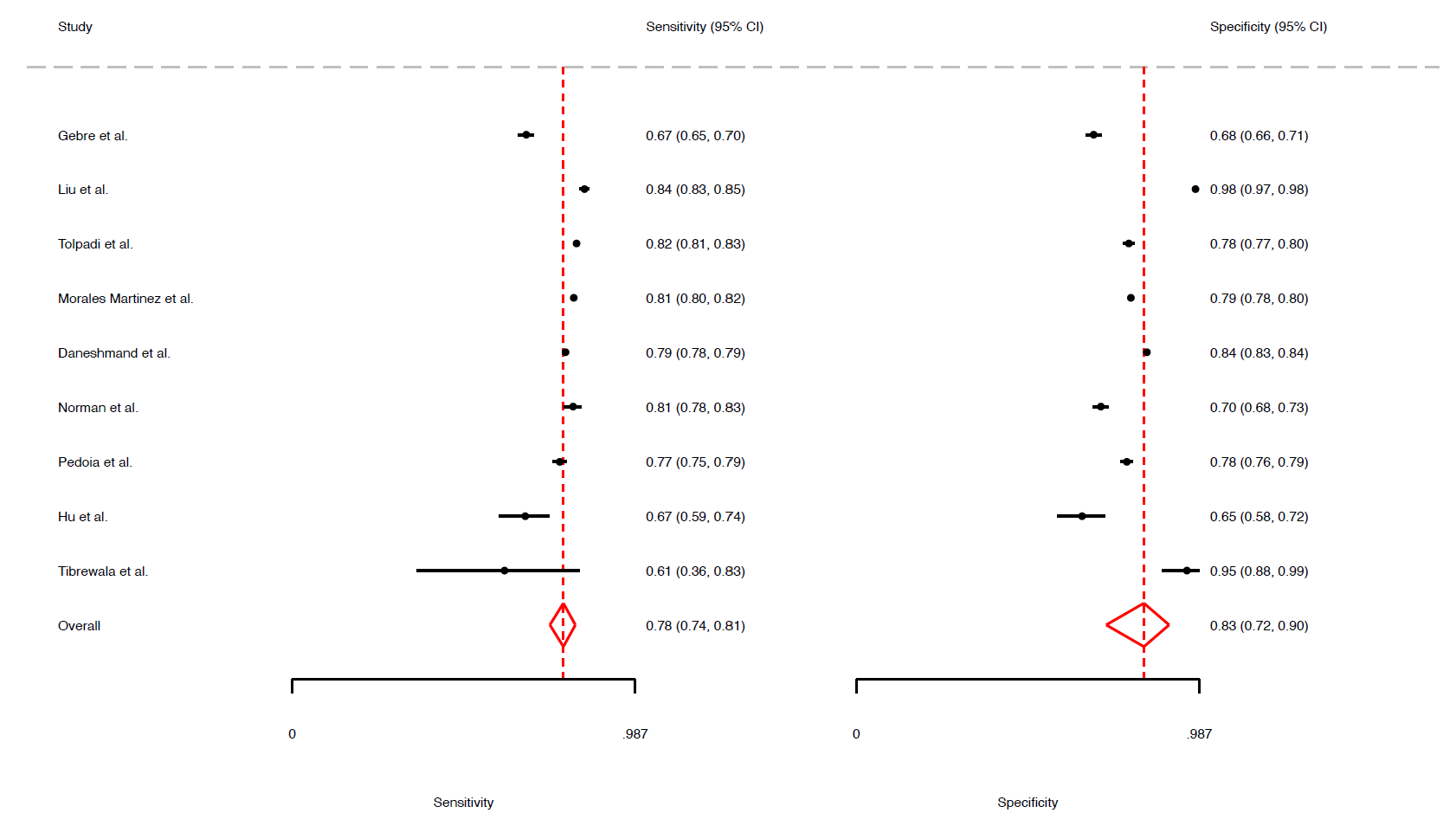


*Meta-Analysis pooled results with estimated sensitivity and specificity values (including 95% confidence intervals) for Deep-Learning only interpretations of cross-sectional imaging.*

**
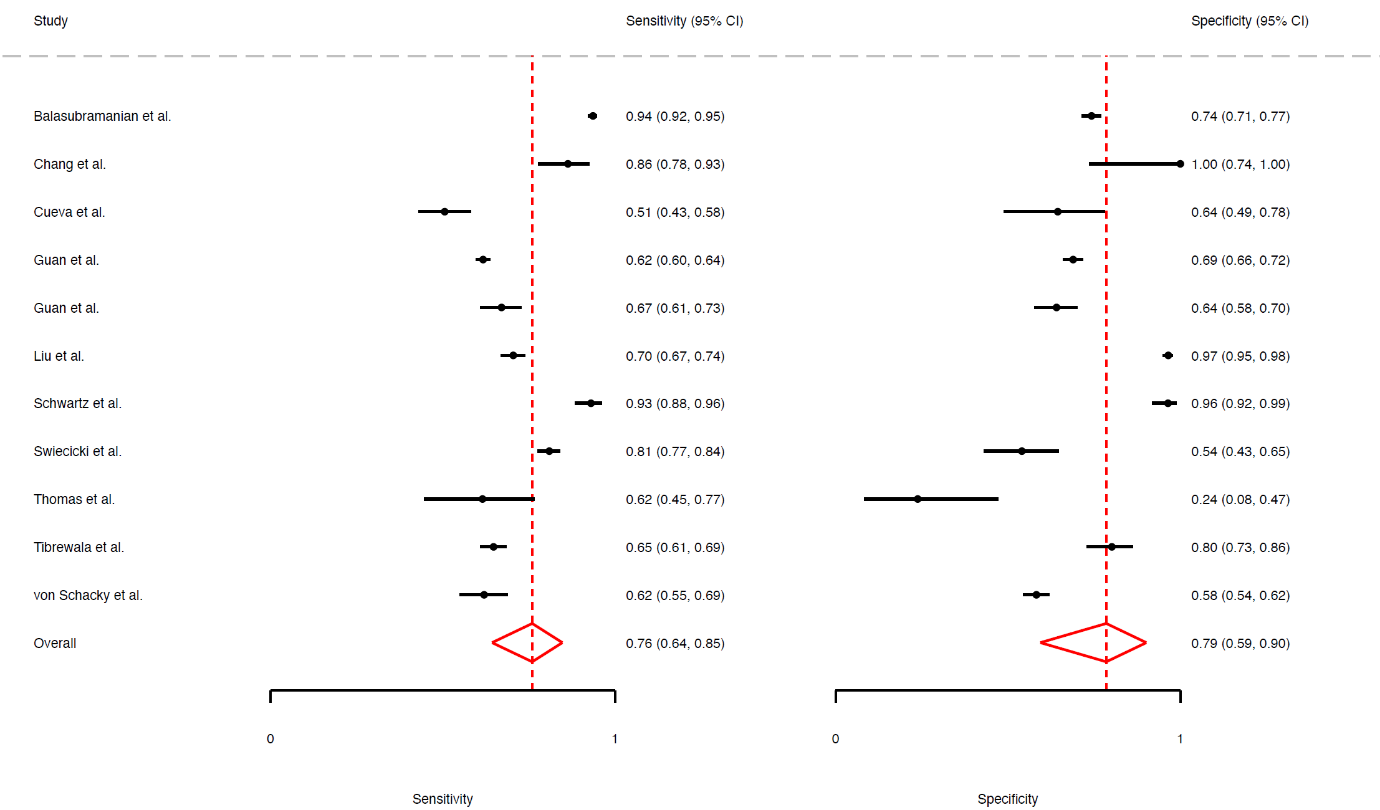
**

*Meta-Analysis pooled results with estimated sensitivity and specificity values (including 95% confidence intervals) for clinician-only interpretations.*

***Tables***

*Summary of the AI and Deep Learning Application studies on the arthroplasty pathway (hip, knee and shoulder) included for analysis****.***

*Abbreviations: ANN: Artificial Neural Networks, CBAM: Convolutional Block Attention Module, CNN: Convolutional Neural Network, DBN: Deep Belief Network, ENN: Ensemble Neural Network, GAE: Graph Auto-Encoder, HRNet: High-Resolution Net, KNN: k-Nearest Neighbors, RF: Radio Frequency, SVM: Support Vector Machine, VGG: Visual Geometry Group, XGB: Extreme Gradient Boosting, YOLO: You Only Look Once,*

| **Study** | | **Year** | **Target Anatomy** | **Algorithm Architecture** | **Category of Study Aim** | **Reference Standard for Ground Truth** | **Imaging Modality** | **Report Sensitivity and Specificity (Y/N)** |
| --- | --- | --- | --- | --- | --- | --- | --- | --- |
| Abdo et al. | | 2022 | Knee | 3D CNN | Disease Classification/Diagnosis | Kellgren Lawrence | X-Ray | N |
| Abedin et al. | | 2019 | Knee | CNN | Disease Classification/Diagnosis | Radiologists interpretation, Kellgren Lawrence | X-Ray | Y |
| Ahmed et al. | | 2023 | Knee | ResNet50 | Disease Classification/Diagnosis | Kellgren Lawrence | X-Ray | N |
| Ahmed et al. | | 2022 | Knee | ResNet50 | Disease Classification/Diagnosis | Kellgren Lawrence | X-Ray | Y |
| Akila et al. | | 2023 | Knee | CNN | Disease Classification/Diagnosis | Kellgren Lawrence | X-Ray | N |
| Al-Rimy et al. | | 2023 | Knee | DenseNet169 | Disease Classification/Diagnosis | Radiologists interpretation, Kellgren Lawrence | X-Ray | N |
| Almansour et al. | | 2023 | Knee | CNN | Disease Classification/Diagnosis | Physician supervised manual labelling | X-Ray | Y |
| Almhdie-Imjabbar et al. | | 2022 | Knee | CNN | Progression Prediction | Kellgren Lawrence, OARSI Grading | X-Ray | N |
| Alzaid et al. | | 2022 | Hip | DenseNet161, ResNet50, Inception, VGG | Disease Classification/Diagnosis | Vancouver Classification System | X-Ray | Y |
| Antonio et al. | | 2022 | Knee | YOLOv3 | Progression Prediction | Kellgren Lawrence | MRI | N |
| Antony et al. | | 2017 | Knee | CNN | Disease Classification/Diagnosis | Kellgren Lawrence | X-Ray | Y |
| Arumugam et al. | | 2022 | Knee | CNN | Progression Prediction | Kellgren Lawrence | X-Ray | N |
| Balasubramanian et al. | | 2022 | Knee | Hybrid DBN | Disease Classification/Diagnosis | Kellgren Lawrence, Radiologists Interpretation | X-Ray | Y |
| Bany Muhammad et al. | | 2019 | Knee | R-CNN | Disease Classification/Diagnosis | Kellgren Lawrence | X-Ray | Y |
| Bayramoglu et al. | | 2020 | Knee | CNN | Disease Classification/Diagnosis | Kellgren Lawrence | X-Ray | N |
| Bayramoglu et al. | | 2022 | Knee | CNN | Disease Classification/Diagnosis | WOMAC scale, Kellgren Lawrence, Radiologists Interpretation | X-Ray | N |
| Borjali et al. | | 2020 | Hip | CNN: DenseNet201 | Post-Operative Complication Detection | Operative Notes | X-Ray | N |
| Chang et al. | | 2020 | Knee | Siamese Neural Network Architecture | Disease Classification/Diagnosis | WOMAC scale, Kellgren Lawrence, Radiologists Interpretation | MRI | N |
| Chaugule et al. | | 2022 | Knee | DenseNet | Disease Classification/Diagnosis | Radiologists interpretation | X-Ray | N |
| Chen et al. | | 2023 | Hip | HipRD and SurgHipNet | Pre-Operative Planning | Surgical Reports, Operative Information | X-Ray | Y |
| Chen et al. | | 2023 | Hip | CNN: SurgHipNet, ResNet101 | Pre-Operative Planning | Surgical reports | X-Ray | Y |
| Chen et al. | | 2022 | Knee | YOLOv3 | Disease Classification/Diagnosis | Kellgren Lawrence, Radiologists Interpretation | X-Ray | N |
| Chen et al. | | 2019 | Knee | YOLOv2, CNN: ResNet, VGG, DenseNet, InceptionV3 | Disease Classification/Diagnosis | Kellgren Lawrence | X-Ray | N |
| Cueva et al. | | 2022 | Knee | Deep Siamese Convolutional Neural Network, ResNet34 | Disease Classification/Diagnosis | Kellgren Lawrence, Radiologists Interpretation | X-Ray | N |
| Daneshmand et al. | | 2024 | Knee | CNN: Unet, ResNet18, ResNet10 | Disease Classification/Diagnosis | OARSI scoring, Radiologists Interpretation | X-Ray and MRI | Y |
| Deniz et al. | | 2022 | Knee | CNN | Disease Classification/Diagnosis | OARSI Scoring, Kellgren Lawrence | CT | Y |
| Dharmani et al. | | 2023 | Knee | CNN: EfficientNetB1 | Disease Classification/Diagnosis | Kellgren Lawrence, Medical Practitioner Interpretation | X-Ray | N |
| Domingues et al. | | 2023 | Knee | CNN: DenseNet161 | Disease Classification/Diagnosis | Kellgren Lawrence, Radiologists Interpretation | X-Ray | Y |
| Tuya et al. | | 2023 | Knee | HRNet | Disease Classification/Diagnosis | Kellgren Lawrence, Radiologists Interpretation | X-Ray | Y |
| En et al. | | 2013 | Knee | ANN | Disease Classification/Diagnosis | Baseline Scans | MRI | N |
| Farajzadeh et al. | | 2023 | Knee | Deep Residual Neural Network: IJES-OA Net | Disease Classification/Diagnosis | Kellgren Lawrence, Radiologists Interpretation | X-Ray | Y |
| Fatema et al. | | 2023 | Knee | KNN, XGB | Disease Classification/Diagnosis | Kellgren Lawrence | X-Ray | Y |
| Feng et al. | | 2021 | Knee | CNN: ResNet | Disease Classification/Diagnosis | Kellgren Lawrence | X-Ray | Y |
| Gebre et al. | | 2022 | Hip | ResNet18 | Disease Classification/Diagnosis | Kellgren Lawrence | X-Ray and CT | Y |
| Geng et al. | | 2023 | Shoulder | CNN: DenseNet121 | Pre-Operative Planning | Operative Notes | X-Ray | Y |
| Guan et al. | | 2020 | Knee | CNN: YOLO, DenseNet | Disease Classification/Diagnosis | Kellgren Lawrence, Radiologists Interpretation | X-Ray | Y |
| Guan et al. | | 2020 | Knee | CNN: YOLO, DenseNet | Disease Progression | WOMAC scale, Kellgren Lawrence, Radiologists Interpretation | X-Ray | Y |
| Guan et al. | | 2022 | Knee | CNN: YOLO, DenseNet, EfficientNet | Disease Progression | WOMAC scale, Kellgren Lawrence, Radiologists Interpretation | X-Ray | Y |
| Guarnera et al. | | 2023 | Hip | CNN: ResNet | Post-Operative Complication Detection | Baseline Images | CT | N |
| Guo et al. | | 2023 | Hip | ResNet18, MC-CSRA | Post-Operative Complication Detection | Orthopaedic Surgeon Interpretation | X-Ray | Y |
| Harish et al. | | 2023 | Knee | CNNs and VGG16 | Disease Classification/Diagnosis | Baseline Radiographic Images | X-Ray | Y |
| Haseeb et al. | | 2023 | Knee | EfficientNet-B0, DenseNet201 | Disease Classification/Diagnosis | Kellgren Lawrence | X-Ray | Y |
| Hema Rajini et al. | | 2023 | Knee | ANN | Disease Classification/Diagnosis | Kellgren Lawrence | X-Ray | N |
| Henson et al. | | 2023 | Elbow | DenseNet121 | Disease Classification/Diagnosis | Medical Expert Interpretation | X-Ray | Y |
| Hill et al. | | 2023 | Knee | CNN: ResNet18 | Disease Progression | KOOS Scale, Kellgren Lawrence | X-Ray | Y |
| Hu et al. | | 2023 | Knee | 3D DenseNet169 | Disease Progression | WOMAC Scale | MRI | Y |
| Hu et al. | | 2022 | Knee | A-ENN | Disease Classification/Diagnosis | Kellgren Lawrence | X-Ray | N |
| Hu et al. | | 2022 | Knee | CNN | Disease Classification/Diagnosis | Arthroscopic Results | MRI | N |
| Jafarzadeh et al. | | 2020 | Knee | CNN: ResNet18, ResNet34, ResNet50, SE-ResNet50, SE-ResNet50-32x4d | Disease Classification/Diagnosis | Kellgren Lawrence, OARSI Scale | X-Ray | N |
| Johny Samuael et al. | | 2023 | Knee | CNN: VGG16, VGG19, MobileNetV2, InceptionV3 | Post-Operative Identification | Senior Orthopaedic Surgeon Interpretation | X-Ray | N |
| Kang et al. | | 2020 | Hip | YOLOv3 | Post-Operative Identification | Manually Labelled Radiographs | X-Ray | N |
| Karnuta et al. | | 2023 | Hip | CNN: Inception V3 | Post-Operative Identification | Surgical Operation Notes | X-Ray | Y |
| Karnuta et al. | | 2023 | Knee | CNN: Inception V3 | Post-Operative Identification | Surgical Operation Notes | X-Ray | Y |
| Khosravi et al. | | 2022 | Hip | EfficientNet-B4, Swin-B, XGBoost | Post-Operative Complication Detection | Surgical Operation Notes | X-Ray | Y |
| Kim et al. | | 2022 | Knee | YOLOv5s | Post-Operative Identification | Experienced Clinician Interpretation | X-Ray | Y |
| Kim et al. | | 2023 | Knee | CNN: VGG19 | Post-Operative Identification | Intraoperative Findings | X-Ray | Y |
| Klemt et al. | | 2022 | Hip and Knee | CNN | Post-Operative Identification | Surgical Operation Notes | X-Ray | Y |
| Kunze et al. | | 2023 | Shoulder | ResNet34 | Post-Operative Identification | Surgical Operation Notes | X-Ray | Y |
| Lau et al. | | 2022 | Knee | Xception | Disease Classification/Diagnosis | Intraoperative Findings, Orthopaedic Surgeon Interpretation | X-Ray | Y |
| Lee et al. | | 2019 | Knee | DenseNet121 | Disease Classification/Diagnosis | KOOS Pain Scores | MRI | N |
| Leung et al. | | 2020 | Knee | CNN: ResNet34 | Disease Classification/Diagnosis | OARSI Scoring, Kellgren Lawrence | X-Ray | Y |
| Li et al. | | 2020 | Knee | R-CNN | Disease Classification/Diagnosis | Kellgren Lawrence | X-Ray | N |
| Liu et al. | | 2020 | Knee | R-CNN | Disease Classification/Diagnosis | Kellgren Lawrence, Radiologists Interpretation | X-Ray | Y |
| Liu et al. | | 2018 | Knee | CNN | Disease Classification/Diagnosis | Radiologist Interpretation | MRI | Y |
| Mahum et al. | | 2021 | Knee | CNN, SVM, KNN, RF | Disease Classification/Diagnosis | Kellgren Lawrence | X-Ray | N |
| Masood et al. | | 2022 | Knee | ResNet152V2, InceptionResNetV2, MobNetV2, EfficientNetB0, EfficientNetB4 | Disease Classification/Diagnosis | Kellgren Lawrence | X-Ray | N |
| Mikhaylichenko et al. | | 2021 | Knee | DenseNet | Disease Classification/Diagnosis | Kellgren Lawrence | X-Ray | N |
| Mishra et al. | | 2024 | Shoulder | CNN: Inception V3, VGG16, Xception, InceptionResNetV2 | Post-Operative Identification | Operative Notes | X-Ray | N |
| Morales Martinez et al. | | 2019 | Knee | CNN | Disease Classification/Diagnosis | Kellgren Lawrence | MRI | Y |
| Nasser et al. | | 2023 | Knee | DST-CNN | Disease Classification/Diagnosis | Kellgren Lawrence | X-Ray | N |
| Nguyen et al. | | 2020 | Knee | Siamese Neural Network Architecture | Disease Classification/Diagnosis | OARSI scoring, Radiologists Interpretation | X-Ray | N |
| Nguyen et al. | | 2020 | Knee | Siamese Neural Network Architecture | Disease Classification/Diagnosis | OARSI Scoring, Kellgren Lawrence | X-Ray | N |
| Norman et al. | | 2019 | Knee | DenseNet | Disease Classification/Diagnosis | Kellgren Lawrence, Radiologists Interpretation | X-Ray | Y |
| Norman et al. | | 2018 | Knee | DenseNet, N-Jet | Disease Classification/Diagnosis | Kellgren Lawrence, Radiologists Interpretation | X-Ray and MRI | Y |
| Olsson et al. | | 2021 | Knee | ResNet | Disease Classification/Diagnosis | Kellgren Lawrence, Radiologists Interpretation | X-Ray | Y |
| Panfilov et al. | | 2022 | Knee | CNN | Disease Progression | Kellgren Lawrence, Radiologists Interpretation | MRI | N |
| Panfilov et al. | | 2020 | Knee | VGG19, Unet | Disease Classification/Diagnosis | WORMS Scoring | MRI | N |
| Pedoia et al. | | 2019 | Knee | DenseNet | Disease Classification/Diagnosis | Kellgren Lawrence | MRI | Y |
| Phan Trung et al. | | 2023 | Knee | GAE | Disease Classification/Diagnosis | Kellgren Lawrence | X-Ray | N |
| Pi et al. | | 2023 | Knee | DenseNet161, EfficientNetb5, EfficientNetV2s, RegNetY8GF, ResNet101, ResNext-50-32x4d, Wide-ResNet-50-2, ShuffleNet-V2-x2-0 | Disease Classification/Diagnosis | Kellgren Lawrence, Radiologists Interpretation | X-Ray | N |
| Pongsakonpruttikul et al. | | 2022 | Knee | YOLOv3 CNN | Disease Classification/Diagnosis | Kellgren Lawrence | X-Ray | Y |
| Rahman et al. | | 2022 | Hip | YOLOv5 | Post-Operative Complication Detection | Radiologists Interpretation | X-Ray | Y |
| Rehman et al. | | 2023 | Knee | CNN | Disease Classification/Diagnosis | Baseline Radiographic Images | X-Ray | N |
| Ribas et al. | 2022 | | Knee | CNN | Disease Classification/Diagnosis | Kellgren Lawrence | X-Ray | Y |
| Rouzrokh et al. | | 2023 | Hip | THA-AID | Disease Classification/Diagnosis | Orthopaedic Surgeon Interpretation | X-Ray | N |
| Rouzrokh et al. | | 2021 | Hip | CNN: YOLOv3, ResNet18 | Progression Prediction | Baseline Radiographic Images | X-Ray | Y |
| Saini et al. | | 2023 | Knee | I-VGG16 | Disease Classification/Diagnosis | Kellgren Lawrence | X-Ray | N |
| Schwartz et al. | | 2020 | Knee | CNN | Disease Classification/Diagnosis | IKDC Scoring, Orthopaedic Surgeon Interpretation | X-Ray | Y |
| Sekhri et al. | | 2023 | Knee | Swin Transformer Model | Disease Classification/Diagnosis | Kellgren Lawrence | X-Ray | N |
| Shah et al. | | 2020 | Knee and Hip | CNN | Post-Operative Complication Detection | Operative Notes | X-Ray | Y |
| Sharma et al. | | 2023 | Knee | EfficientNetB5 | Disease Classification/Diagnosis | Kellgren Lawrence, Radiologists Interpretation | X-Ray | Y |
| Sharma et al. | | 2021 | Knee | DenseNet201 | Pre-Operative Planning | Manually Labelled Radiographs | X-Ray | Y |
| Sindhu et al. | | 2022 | Knee | CNN | Progression Prediction | Kellgren Lawrence | X-Ray | Y |
| Sivakumari et al. | | 2022 | Knee | AlexNet | Disease Classification/Diagnosis | Kellgren Lawrence | X-Ray | N |
| Sivakumari et al. | | 2024 | Knee | R-CNN | Disease Classification/Diagnosis | Baseline Radiographic Images | X-Ray | N |
| Suresha et al. | | 2018 | Knee | R-CNN | Disease Classification/Diagnosis | Kellgren Lawrence | X-Ray | N |
| Swiecicki et al. | | 2021 | Knee | VGG16, R-CNN | Disease Classification/Diagnosis | Kellgren Lawrence, Radiologists Interpretation | X-Ray | N |
| Tariq et al. | | 2023 | Knee | CNN: ResNet34, VGG19, DenseNet121, DenseNet161 | Disease Classification/Diagnosis | Kellgren Lawrence | X-Ray | N |
| Thomas et al. | | 2020 | Knee | CNN | Disease Classification/Diagnosis | Kellgren Lawrence, Radiologists Interpretation | X-Ray | Y |
| Tibrewala et al. | | 2020 | Hip | MRNet | Disease Classification/Diagnosis | SHOMRI Scoring, Radiologists Interpretation | MRI | Y |
| Tibrewala et al. | | 2019 | Hip | DCNN | Disease Classification/Diagnosis | Radiologists Interpretation | MRI | Y |
| Tiulpin et al. | | 2019 | Knee | SE-ResNetXt 50 32x4d | Disease Classification/Diagnosis | Kellgren Lawrence | X-Ray | N |
| Tiulpin et al. | | 2019 | Knee | CNN | Disease Classification/Diagnosis | Kellgren Lawrence, WOMAC Scoring | X-Ray | N |
| Tiulpin et al. | | 2019 | Knee | Deep Siamese CNN | Disease Classification/Diagnosis | Kellgren Lawrence, Radiologists Interpretation | X-Ray | N |
| Tiulpin et al. | | 2020 | Knee | CNN: SE-ResNet50, SE-ResNext50-32x4d | Disease Classification/Diagnosis | OARSI Grading, Kellgren Lawrence, Radiologists Interpretation | X-Ray | N |
| Tiulpin et al. | | 2018 | Knee | CNN | Disease Classification/Diagnosis | Kellgren Lawrence, Clinical Expert Interpretation | X-Ray | N |
| Tiwari et al. | | 2022 | Knee | ResNet50, VGG-16, InceptionV3, MobilNetV2, EfficientNetB7, DenseNet201, Xception, NasNetMobile | Disease Classification/Diagnosis | Kellgren Lawrence, Orthopaedic Surgeon Interpretation | X-Ray | N |
| Tiwari et al. | | 2022 | Knee | VGG-16, ResNet50, MobileNet, EfficientNetB7, InceptionV3, NasNet, Xception | Post-Operative Identification | Operative Notes | X-Ray | N |
| Tolpadi et al. | | 2020 | Knee | DenseNet121 | Progression Prediction | Kellgren Lawrence | MRI | Y |
| Upadhyay et al. | | 2023 | Knee | CNN | Disease Classification/Diagnosis | Kellgren Lawrence | X-Ray | N |
| Urban et al. | | 2020 | Shoulder | CNN: VGG16, VGG19, ResNet50, DenseNet, NASNet | Post-Operative Identification | Baseline Radiographic Images | X-Ray | N |
| Ureten et al. | | 2020 | Hip | CNN: VGG16 | Disease Classification/Diagnosis | Radiologist Interpretation | X-Ray | Y |
| Vijaya et al. | | 2023 | Knee | CNN | Disease Classification/Diagnosis | Kellgren Lawrence, Radiologists Interpretation | X-Ray | N |
| Vijaya et al. | | 2023 | Knee | CNN: VGG16 | Disease Classification/Diagnosis | Kellgren Lawrence | X-Ray | Y |
| von Schacky et al. | | 2019 | Hip | DenseNet161 | Disease Classification/Diagnosis | OARSI Grading, Radiologists Interpretation | X-Ray | Y |
| von Schacky et al. | | 2019 | Hip | DenseNet161 | Disease Classification/Diagnosis | OARSI Grading | X-Ray | N |
| von Schacky et al. | | 2020 | Hip | DenseNet161 | Disease Classification/Diagnosis | OARSI Grading, Radiologists Interpretation | X-Ray | Y |
| von Schacky et al. | | 2020 | Hip | DenseNet161 | Disease Classification/Diagnosis | OARSI Grading, Radiologists Interpretation | X-Ray | Y |
| Wahyuningrum et al. | | 2019 | Knee | CNN: VGG16 | Disease Classification/Diagnosis | Kellgren Lawrence | X-Ray | N |
| Wang et al. | | 2021 | Knee | CNN | Disease Classification/Diagnosis | Kellgren Lawrence | X-Ray | N |
| Wang et al. | | 2022 | Knee | CNN | Disease Classification/Diagnosis | Kellgren Lawrence | X-Ray | N |
| Wang et al. | | 2023 | Knee | CNN | Disease Classification/Diagnosis | Kellgren Lawrence, Radiologists Interpretation | X-Ray | N |
| Wang et al. | | 2022 | Knee | ResNet50 | Disease Classification/Diagnosis | Kellgren Lawrence, Radiologists Interpretation | X-Ray | Y |
| Wang et al. | | 2022 | Knee | JC-RegNet | Disease Classification/Diagnosis | Baseline Radiographic Images | X-Ray | N |
| Wei et al. | | 2022 | Hip | CNN: ResNet152, InceptionV3, DenseNet161, ResNeXt101, Wide-ResNet101, RedNetX-32GF | Disease Classification/Diagnosis | Baseline Radiographic Images | X-Ray | Y |
| Xu et al. | | 2023 | Hip | DCNN: EfficientNetB4 | Disease Classification/Diagnosis | Kellgren Lawrence, Radiologists Interpretation | X-Ray | Y |
| Yan et al. | | 2020 | Knee | CNN: DenseNet, Inception | Post-Operative Identification | Baseline Radiographic Images | X-Ray | N |
| Yang et al. | | 2022 | Knee | RefineDet | Disease Classification/Diagnosis | Kellgren Lawrence, Orthopaedic Surgeon Interpretation | X-Ray | Y |
| Yassine et al. | | 2022 | Knee | CNN DenseNet | Disease Classification/Diagnosis | Kellgren Lawrence, Radiologists Interpretation | X-Ray | N |
| Yeoh et al. | | 2023 | Knee | 3D CNN: ResNet, DenseNet, VGG, AlexNet | Disease Classification/Diagnosis | Kellgren Lawrence | MRI | N |
| Yi et al. | | 2020 | Shoulder | DCNN: ResNet18, ResNet152 | Disease Classification/Diagnosis | Radiologists Interpretation | X-Ray | Y |
| Yi et al. | | 2020 | Knee | DCNNL ResNet18, ResNet152 | Disease Classification/Diagnosis | Radiologists Interpretation | X-Ray | Y |
| Yin et al. | | 2024 | Knee | BikNet | Disease Classification/Diagnosis | Kellgren Lawrence, Clinical Expert Interpretation | X-Ray | Y |
| Yoon et al. | | 2023 | Knee | HRNet, RetinaNet, NASNet | Disease Classification/Diagnosis | Kellgren Lawrence, Radiologists Interpretation | X-Ray | N |
| Yue et al. | | 2019 | Knee | CNN: ResNet18 | Disease Classification/Diagnosis | Radiologists Interpretation | X-Ray | N |
| Yuniarno et al. | | 2022 | Knee | DCNN | Disease Classification/Diagnosis | Kellgren Lawrence | X-Ray | N |
| **Yunus et al.** | | 2022 | Knee | YOLOv2 | Disease Classification/Diagnosis | Kellgren Lawrence, Radiologists Interpretation | X-Ray | Y |
| Zhang et al. | | 2020 | Knee | ResNet34, CBAM | Disease Classification/Diagnosis | Kellgren Lawrence | X-Ray | N |

*Statistical performance of AI and Deep Learning Applications in the arthroplasty pathway (hip, knee and shoulder) analysed for all included studies.*

| **Study** | **Year** | **Specificity** | **Sensitivity** | **Area Under The Curve** | **Precision** | **Average Precision** | **F1 Score** | **Jaccard Index** | **Dice Coefficient** | **Classification** |
| --- | --- | --- | --- | --- | --- | --- | --- | --- | --- | --- |
| Abdo et al. | 2022 | N/A | N/A | 0.92 | N/A | N/A | N/A | N/A | N/A | Diagnostic Imaging |
| Abedin et al. | 2019 | N/A | N/A | N/A | N/A | N/A | N/A | N/A | N/A | Diagnostic Imaging |
| Ahmed et al. | 2023 | N/A | N/A | N/A | N/A | N/A | N/A | N/A | N/A | Diagnostic Imaging |
| Ahmed et al. | 2022 | 0.89 | 0.59 | N/A | N/A | N/A | N/A | N/A | N/A | Diagnostic Imaging |
| Akila et al. | 2023 | N/A | N/A | N/A | N/A | N/A | N/A | N/A | N/A | Diagnostic Imaging |
| Al-Rimy et al. | 2023 | N/A | N/A | N/A | 0.83 | N/A | 0.821 | N/A | N/A | Diagnostic Imaging |
| Almansour et al. | 2023 | 0.97 | 0.99 | N/A | 0.98 | N/A | 0.98 | N/A | N/A | Diagnostic Imaging |
| Almhdie-Imjabbar et al. | 2022 | N/A | N/A | 0.78 | N/A | N/A | N/A | N/A | N/A | Prognostic Imaging |
| Alzaid et al. | 2022 | 0.95 | 0.88 | N/A | 0.8 | N/A | 0.94 | N/A | N/A | Diagnostic Imaging |
| Antonio et al. | 2022 | N/A | N/A | N/A | 0.99 | 0.97 | N/A | N/A | N/A | Prognostic Imaging |
| Antony et al. | 2017 | 0.87 | 0.72 | 0.86 | 0.605 | N/A | 0.57 | 0.83 | N/A | Diagnostic Imaging |
| Arumugam et al. | 2022 | N/A | N/A | N/A | N/A | N/A | N/A | N/A | N/A | Prognostic Imaging |
| Balasubramanian et al. | 2022 | 0.94 | 0.98 | N/A | N/A | N/A | N/A | N/A | N/A | Diagnostic Imaging |
| Bany Muhammad et al. | 2019 | N/A | N/A | N/A | N/A | N/A | N/A | 0.91 | N/A | Diagnostic Imaging |
| Bayramoglu et al. | 2020 | N/A | N/A | 0.95 | N/A | N/A | N/A | N/A | N/A | Diagnostic Imaging |
| Bayramoglu et al. | 2022 | N/A | N/A | 0.89 | N/A | 0.714 | N/A | N/A | N/A | Diagnostic Imaging |
| Borjali et al. | 2020 | N/A | N/A | N/A | N/A | N/A | N/A | N/A | N/A | Prognostic Imaging |
| Chang et al. | 2020 | N/A | N/A | 0.83 | N/A | N/A | N/A | N/A | N/A | Diagnostic Imaging |
| Chaugule et al. | 2022 | N/A | N/A | N/A | N/A | N/A | N/A | N/A | N/A | Diagnostic Imaging |
| Chen et al. | 2023 | 1 | 0.95 | 0.97 | 1 | N/A | 0.97 | N/A | N/A | Pre-Operative Planning |
| Chen et al. | 2023 | 0.92 | 0.93 | 0.99 | N/A | N/A | 0.94 | N/A | N/A | Pre-Operative Planning |
| Chen et al. | 2022 | 0.79 | 0.29 | N/A | 0.76 |  | 0.76 | N/A | N/A | Diagnostic Imaging |
| Chen et al. | 2019 | N/A | N/A | N/A | N/A | N/A | N/A | 0.89 | N/A | Diagnostic Imaging |
| Cueva et al. | 2022 | 0.7 | 0.59 | N/A | 0.67 | N/A | 0.61 | N/A | N/A | Diagnostic Imaging |
| Daneshmand et al. | 2024 | 0.84 | 0.163 | 0.81 | 0.80 | 0.80 | N/A | N/A | N/A | Diagnostic Imaging |
| Deniz et al. | 2022 | 0.64 | 0.708 | 0.7 | N/A | N/A | N/A | N/A | N/A | Diagnostic Imaging |
| Dharmani et al. | 2023 | 1 | 0.943 | N/A | 0.89 | N/A | 0.89 | N/A | N/A | Diagnostic Imaging |
| Domingues et al. | 2023 | 0.76 | 0.821 | 0.87 | N/A | N/A | 0.55 | N/A | N/A | Diagnostic Imaging |
|  | 2023 | 0.95 | 0.81 | 0.91 | N/A | N/A | 0.86 | N/A | N/A | Diagnostic Imaging |
| En et al. | 2013 | N/A | N/A | N/A | N/A | N/A | N/A | N/A | N/A | Diagnostic Imaging |
| Farajzadeh et al. | 2023 | 0.74 | 0.8 | N/A | 0.79 | N/A | 0.80 | N/A | N/A | Diagnostic Imaging |
| Fatema et al. | 2023 | 0.99 | 0.99 | N/A | 0.99 | N/A | 0.99 | N/A | N/A | Diagnostic Imaging |
| Feng et al. | 2021 | 0.67 | 0.33 | N/A | 0.70 | N/A | 0.68 | N/A | N/A | Diagnostic Imaging |
| Gebre et al. | 2022 | 0.68 | 0.82 | 0.91 | 0.82 | N/A | 0.82 | N/A | N/A | Diagnostic Imaging |
| Geng et al. | 2023 | 0.89 | 0.83 | N/A | 0.94 | N/A | 0.94 | N/A | N/A | Pre-Operative Planning |
| Guan et al. | 2020 | 0.76 | 0.78 | 0.80 | N/A | 0.94 | N/A | N/A | N/A | Diagnostic Imaging |
| Guan et al. | 2020 | 0.74 | 0.66 | 0.75 | N/A | N/A | N/A | N/A | N/A | Diagnostic Imaging |
| Guan et al. | 2022 | 0.71 | 0.77 | 0.77 | N/A | N/A | N/A | N/A | N/A | Diagnostic Imaging |
| Guarnera et al. | 2023 | N/A | N/A | N/A | N/A | N/A | N/A | N/A | N/A | Prognostic Imaging |
| Guo et al. | 2023 | N/A | 0.93 | N/A | 0.57 | 0.35 | 0.43 | N/A | N/A | Prognostic Imaging |
| Harish et al. | 2023 | N/A | 0.9 | N/A | 0.91 | N/A | 0.91 | N/A | N/A | Diagnostic Imaging |
| Haseeb et al. | 2023 | N/A | 0.9 | N/A | 0.88 | N/A | 0.89 | N/A | N/A | Diagnostic Imaging |
| Hema Rajini et al. | 2023 | N/A | N/A | N/A | N/A | N/A | N/A | N/A | N/A | Diagnostic Imaging |
| Henson et al. | 2023 | 0.98 | 0.93 | N/A | 0.98 | N/A | 0.96 | N/A | N/A | Diagnostic Imaging |
| Hill et al. | 2023 | 0.61 | 0.62 | 0.78 | N/A | N/A | N/A | N/A | N/A | Prognostic Imaging |
| Hu et al. | 2023 | 0.70 | 0.63 | 0.73 | N/A | N/A | N/A | N/A | N/A | Prognostic Imaging |
| Hu et al. | 2022 | N/A | N/A | N/A | N/A | N/A | N/A | N/A | N/A | Diagnostic Imaging |
| Hu et al. | 2022 | N/A | N/A | N/A | N/A | N/A | N/A | N/A | N/A | Diagnostic Imaging |
| Jafarzadeh et al. | 2020 | N/A | N/A | 0.88 | N/A | N/A | N/A | N/A | N/A | Diagnostic Imaging |
| Johny Samuael et al. | 2023 | N/A | N/A | 0.91 | 0.80 | N/A | 0.72 | N/A | N/A | Prognostic Imaging |
| Kang et al. | 2020 | N/A | N/A | 0.99 | 0.99 | N/A | N/A | N/A | N/A | Prognostic Imaging |
| Karnuta et al. | 2023 | 0.99 | 0.69 | 0.99 | N/A | N/A | N/A | N/A | N/A | Prognostic Imaging |
| Karnuta et al. | 2023 | 0.99 | 0.95 | 0.99 | 0.941 | N/A | N/A | N/A | N/A | Prognostic Imaging |
| Khosravi et al. | 2022 | 0.68 | 0.69 | 0.77 | 0.1 | N/A | N/A | N/A | N/A | Prognostic Imaging |
| Kim et al. | 2022 | 0.99 | 0.69 | N/A | N/A | N/A | N/A | N/A | N/A | Prognostic Imaging |
| Kim et al. | 2023 | 0.99 | 0.95 | N/A | 0.98 | N/A | N/A | N/A | N/A | Prognostic Imaging |
| Klemt et al. | 2022 | 0.98 | 0.95 | 0.97 | N/A | N/A | N/A | N/A | N/A | Prognostic Imaging |
| Kunze et al. | 2023 | N/A | 0.9 | 0.99 | N/A | N/A | N/A | N/A | N/A | Prognostic Imaging |
| Lau et al. | 2022 | 0.91 | 0.96 | 0.94 | 0.92 | N/A | N/A | N/A | N/A | Diagnostic Imaging |
| Lee et al. | 2019 | N/A | N/A | N/A | N/A | N/A | N/A | N/A | N/A | Diagnostic Imaging |
| Leung et al. | 2020 | 0.77 | 0.83 | 0.87 | N/A | N/A | N/A | N/A | N/A | Diagnostic Imaging |
| Li et al. | 2020 | N/A | N/A | N/A | N/A | N/A | N/A | N/A | N/A | Diagnostic Imaging |
| Liu et al. | 2020 | 0.94 | 0.78 | 0.82 | 0.82 | 0.82 | 0.79 | 0.92 | 0.96 | Diagnostic Imaging |
| Liu et al. | 2018 | 0.87 | 0.82 | 0.92 | N/A | N/A | N/A | N/A | 0.82 | Diagnostic Imaging |
| Mahum et al. | 2021 | N/A | N/A | N/A | 0.98 | N/A | N/A | N/A | N/A | Diagnostic Imaging |
| Masood et al. | 2022 | N/A | N/A | 0.89 | N/A | N/A | N/A | N/A | N/A | Diagnostic Imaging |
| Mikhaylichenko et al. | 2021 | N/A | N/A | N/A | N/A | N/A | N/A | 0.75 | N/A | Diagnostic Imaging |
| Mishra et al. | 2024 | N/A | N/A | 0.99 | 0.83 | N/A | 0.82 | N/A | N/A | Diagnostic Imaging |
| Morales Martinez et al. | 2019 | 0.79 | 0.81 | N/A | N/A | N/A | N/A | N/A | N/A | Diagnostic Imaging |
| Nasser et al. | 2023 | N/A | N/A | 0.83 | 0.65 | N/A | 0.64 | N/A | N/A | Diagnostic Imaging |
| Nguyen et al. | 2020 | N/A | N/A | N/A | N/A | N/A | N/A | N/A | N/A | Diagnostic Imaging |
| Nguyen et al. | 2020 | N/A | N/A | N/A | N/A | N/A | N/A | N/A | N/A | Diagnostic Imaging |
| Norman et al. | 2019 | 0.991 | 0.86 | N/A | N/A | N/A | N/A | N/A | N/A | Diagnostic Imaging |
| Norman et al. | 2018 | 0.70 | 0.30 | 0.9 | N/A | N/A | N/A | N/A | N/A | Diagnostic Imaging |
| Olsson et al. | 2021 | 0.88 | 0.82 | 0.88 | N/A | N/A | N/A | N/A | N/A | Diagnostic Imaging |
| Panfilov et al. | 2022 | N/A | N/A | 0.79 | 0.58 | 0.58 | N/A | N/A | N/A | Prognostic Imaging |
| Panfilov et al. | 2020 | N/A | N/A | N/A | N/A | N/A | N/A | N/A | N/A | Diagnostic Imaging |
| Pedoia et al. | 2019 | 0.80 | 0.77 | 0.82 | N/A | N/A | N/A | N/A | N/A | Diagnostic Imaging |
| Phan Trung et al. | 2023 | N/A | N/A | N/A | N/A | N/A | N/A | N/A | N/A | Diagnostic Imaging |
| Pi et al. | 2023 | 0.89 | 0.80 | N/A | 0.79 | N/A | 0.77 | N/A | N/A | Diagnostic Imaging |
| Pongsakonpruttikul et al. | 2022 | 0.85 | 0.85 | 0.77 | 0.85 | 0.81 | 0.85 | N/A | N/A | Diagnostic Imaging |
| Rahman et al. | 2022 | 0.95 | 0.95 | 0.98 | 0.95 | 0.88 | 0.95 | N/A | N/A | Prognostic Imaging |
| Rehman et al. | 2023 | N/A | N/A | N/A | 0.77 | N/A | 0.81 | N/A | N/A | Diagnostic Imaging |
| Ribas et al. | 2022 | 0.86 | 0.78 | N/A | N/A | N/A | N/A | N/A | N/A | Diagnostic Imaging |
| Rouzrokh et al. | 2023 | N/A | N/A | 0.97 | N/A | N/A | 0.99 | N/A | N/A | Diagnostic Imaging |
| Rouzrokh et al. | 2021 | 0.49 | 0.89 | 0.77 | N/A | N/A | N/A | N/A | N/A | Prognostic Imaging |
| Saini et al. | 2023 | N/A | N/A | N/A | 0.71 | N/A | 0.7 | N/A | N/A | Diagnostic Imaging |
| Schwartz et al. | 2020 | 0.91 | 0.97 | 0.94 | N/A | N/A | N/A | N/A | N/A | Diagnostic Imaging |
| Sekhri et al. | 2023 | N/A | N/A | N/A | N/A | N/A | 0.69 | N/A | N/A | Diagnostic Imaging |
| Shah et al. | 2020 | 0.96 | 0.70 | N/A | 0.87 | N/A | N/A | N/A | N/A | Prognostic Imaging |
| Sharma et al. | 2023 | 0.97 | 0.81 | N/A | 0.87 | N/A | 0.86 | N/A | N/A | Diagnostic Imaging |
| Sharma et al. | 2021 | N/A | 0.97 | 0.99 | N/A | N/A | N/A | N/A | N/A | Pre-Operative Planning |
| Sindhu et al. | 2022 | 0.78 | 0.82 | 0.87 | N/A | N/A | N/A | N/A | N/A | Prognostic Imaging |
| Sivakumari et al. | 2022 | N/A | N/A | N/A | N/A | N/A | N/A | N/A | N/A | Diagnostic Imaging |
| Sivakumari et al. | 2024 | N/A | N/A | N/A | N/A | N/A | N/A | N/A | N/A | Diagnostic Imaging |
| Suresha et al. | 2018 | N/A | N/A | N/A | N/A | N/A | N/A | N/A | N/A | Diagnostic Imaging |
| Swiecicki et al. | 2021 | 0.73 | 0.92 | N/A | N/A | N/A | N/A | N/A | N/A | Diagnostic Imaging |
| Tariq et al. | 2023 | N/A | N/A | N/A | 0.98 | N/A | 0.97 | N/A | N/A | Diagnostic Imaging |
| Thomas et al. | 2020 | 0.92 | 0.80 | N/A | 0.68 | N/A | 0.7 | N/A | N/A | Diagnostic Imaging |
| Tibrewala et al. | 2020 | N/A | N/A | 0.81 | N/A | N/A | N/A | N/A | N/A | Diagnostic Imaging |
| Tibrewala et al. | 2019 | 0.8 | 0.7 | N/A | N/A | N/A | N/A | N/A | N/A | Diagnostic Imaging |
| Tiulpin et al. | 2019 | N/A | N/A | 0.71 | N/A | N/A | N/A | N/A | N/A | Diagnostic Imaging |
| Tiulpin et al. | 2019 | N/A | N/A | 0.79 | 0.68 | 0.68 | N/A | N/A | N/A | Diagnostic Imaging |
| Tiulpin et al. | 2019 | N/A | N/A | 0.8 | N/A | N/A | N/A | N/A | N/A | Diagnostic Imaging |
| Tiulpin et al. | 2020 | N/A | N/A | 0.98 | 0.98 | N/A | N/A | N/A | N/A | Diagnostic Imaging |
| Tiulpin et al. | 2018 | 0.78 | 0.76 | 0.93 | N/A | N/A | N/A | N/A | N/A | Diagnostic Imaging |
| Tiwari et al. | 2022 | N/A | N/A | N/A | 0.94 | N/A | N/A | N/A | N/A | Diagnostic Imaging |
| Tiwari et al. | 2022 | N/A | 0.94 | N/A | 0.98 | N/A | N/A | N/A | N/A | Diagnostic Imaging |
| Tolpadi et al. | 2020 | 0.78 | 0.82 | 0.83 | N/A | N/A | N/A | N/A | N/A | Prognostic Imaging |
| Upadhyay et al. | 2023 | N/A | N/A | N/A | N/A | N/A | N/A | N/A | N/A | Diagnostic Imaging |
| Urban et al. | 2020 | N/A | N/A | 0.75 | N/A | N/A | 0.69 | N/A | N/A | Post-Operative Identification |
| Ureten et al. | 2020 | 0.83 | 0.98 | N/A | 0.85 | N/A | N/A | N/A | N/A | Diagnostic Imaging |
| V et al. | 2023 | N/A | N/A | N/A | N/A | N/A | N/A | N/A | N/A | Diagnostic Imaging |
| Vijaya et al. | 2023 | 0.95 | 0.98 | 0.95 | N/A | N/A | N/A | N/A | N/A | Diagnostic Imaging |
| von Schacky et al. | 2019 | 0.48 | 0.94 | 0.72 | N/A | N/A | N/A | N/A | N/A | Diagnostic Imaging |
| von Schacky et al. | 2019 | N/A | N/A | 0.9 | N/A | N/A | N/A | N/A | N/A | Diagnostic Imaging |
| von Schacky et al. | 2020 | 0.99 | 0.84 | 0.92 | N/A | N/A | N/A | N/A | N/A | Diagnostic Imaging |
| von Schacky et al. | 2020 | 0.91 | 0.60 | 0.91 | 0.88 | N/A | 0.87 | N/A | N/A | Diagnostic Imaging |
| Wahyuningrum et al. | 2019 | N/A | N/A | N/A | N/A | N/A | N/A | N/A | N/A | Diagnostic Imaging |
| Wang et al. | 2021 | N/A | N/A | N/A | N/A | N/A | N/A | N/A | N/A | Diagnostic Imaging |
| Wang et al. | 2022 | N/A | N/A | N/A | N/A | N/A | N/A | N/A | N/A | Diagnostic Imaging |
| Wang et al. | 2023 | 0.891 | 0.96 | N/A | N/A | N/A | 0.91 | N/A | N/A | Diagnostic Imaging |
| Wang et al. | 2022 | 0.859 | 0.96 | N/A | 0.81 | N/A | 0.81 | 0.80 | N/A | Diagnostic Imaging |
| Wang et al. | 2022 | N/A | N/A | N/A | N/A | N/A | 0.86 | N/A | N/A | Diagnostic Imaging |
| Wei et al. | 2022 | 1 | 1 | 0.99 | N/A | N/A | N/A | N/A | N/A | Diagnostic Imaging |
| Xu et al. | 2023 | 0.87 | 0.93 | 0.94 | N/A | N/A | N/A | N/A | N/A | Diagnostic Imaging |
| Yan et al. | 2020 | N/A | N/A | N/A | 0.97 | N/A | 0.98 | N/A | N/A | Diagnostic Imaging |
| Yang et al. | 2022 | 0.99 | 0.98 | 0.98 | 0.87 | N/A | 0.91 | N/A | N/A | Diagnostic Imaging |
| Yassine et al. | 2022 | N/A | N/A | N/A | 0.88 | N/A | 0.87 | N/A | N/A | Diagnostic Imaging |
| Yeoh et al. | 2023 | N/A | N/A | 0.92 | 0.85 | N/A | 0.82 | N/A | N/A | Diagnostic Imaging |
| Yi et al. | 2020 | 0.97 | 0.9 | 0.94 | N/A | N/A | N/A | N/A | N/A | Diagnostic Imaging |
| Yi et al. | 2020 | 1 | 1 | N/A | N/A | N/A | N/A | N/A | N/A | Diagnostic Imaging |
| Yin et al. | 2024 | 0.76 | 0.67 | 0.75 | N/A | N/A | N/A | N/A | N/A | Diagnostic Imaging |
| Yoon et al. | 2023 | 0.81 | 1 | N/A | 0.82 | N/A | 0.83 | N/A | N/A | Diagnostic Imaging |
| Yue et al. | 2019 | N/A | N/A | N/A | N/A | N/A | N/A | N/A | N/A | Diagnostic Imaging |
| Yuniarno et al. | 2022 | N/A | N/A | N/A | N/A | N/A | N/A | N/A | N/A | Diagnostic Imaging |
| Yunus et al. | 2022 | 0.95 | 0.84 | N/A | 0.82 | N/A | 0.88 | N/A | N/A | Diagnostic Imaging |
| Zhang et al. | 2020 | N/A | N/A | N/A | N/A | N/A | N/A | N/A | N/A | Diagnostic Imaging |

*Statistical performance of clinician interpretation in the arthroplasty pathway (hip, knee and shoulder) analysed for all included studies.*

| **Study** | **Year** | **Specificity** | **Sensitivity** | **Area Under The Curve** | **Precision** | **Average Precision** | **F1 Score** | **Jaccard Index** | **Dice Coefficient** | **Classification** |
| --- | --- | --- | --- | --- | --- | --- | --- | --- | --- | --- |
| Abedin et al. | 2019 | N/A | N/A | N/A | N/A | N/A | N/A | N/A | N/A | N/A |
| Al-Rimy et al. | 2023 | N/A | N/A | N/A | N/A | N/A | N/A | N/A | N/A | N/A |
| Almansour et al. | 2023 | N/A | N/A | N/A | N/A | N/A | N/A | N/A | N/A | N/A |
| Balasubramanian et al. | 2022 | 0.74 | 0.94 | N/A | N/A | N/A | N/A | N/A | N/A | N/A |
| Bayramoglu et al. | 2022 | N/A | N/A | 0.82 | N/A | 0.49 | N/A | N/A | N/A | N/A |
| Chang et al. | 2020 | 1 | 0.86 | N/A | N/A | N/A | N/A | N/A | N/A | N/A |
| Chen et al. | 2023 | N/A | N/A | N/A | N/A | N/A | N/A | N/A | N/A | N/A |
| Chen et al. | 2022 | N/A | N/A | N/A | N/A | N/A | N/A | N/A | N/A | N/A |
| Cueva et al. | 2022 | 0.64 | 0.51 | N/A | 0.57 | N/A | N/A | N/A | N/A | N/A |
| Daneshmand et al. | 2024 | N/A | N/A | N/A | N/A | N/A | N/A | N/A | N/A | N/A |
| Dharmani et al. | 2023 | N/A | N/A | N/A | N/A | N/A | N/A | N/A | N/A | N/A |
| Domingues et al. | 2023 | N/A | N/A | N/A | N/A | N/A | N/A | N/A | N/A | N/A |
| ETuya et al. | 2023 | N/A | N/A | N/A | N/A | N/A | N/A | N/A | N/A | N/A |
| Farajzadeh et al. | 2023 | N/A | N/A | N/A | N/A | N/A | N/A | N/A | N/A | N/A |
| Guan et al. | 2020 | N/A | N/A | N/A | N/A | N/A | N/A | N/A | N/A | N/A |
| Guan et al. | 2020 | 0.69 | 0.62 | 0.64 | N/A | N/A | N/A | N/A | N/A | N/A |
| Guan et al. | 2022 | 0.64 | 0.67 | 0.69 | N/A | N/A | N/A | N/A | N/A | N/A |
| Guo et al. | 2023 | N/A | N/A | N/A | N/A | N/A | N/A | N/A | N/A | N/A |
| Henson et al. | 2023 | N/A | N/A | N/A | N/A | N/A | N/A | N/A | N/A | N/A |
| Kim et al. | 2022 | N/A | N/A | N/A | N/A | N/A | N/A | N/A | N/A | N/A |
| Lau et al. | 2022 | 0.95 | 0.95 | N/A | N/A | N/A | N/A | N/A | N/A | N/A |
| Liu et al. | 2020 | N/A | N/A | N/A | N/A | N/A | N/A | N/A | N/A | N/A |
| Liu et al. | 2018 | 0.97 | 0.71 | N/A | N/A | N/A | N/A | N/A | N/A | N/A |
| Nguyen et al. | 2020 | N/A | N/A | N/A | N/A | N/A | N/A | N/A | N/A | N/A |
| Norman et al. | 2019 | 0.91 | N/A | N/A | N/A | N/A | N/A | N/A | N/A | N/A |
| Norman et al. | 2018 | N/A | N/A | N/A | N/A | N/A | N/A | N/A | N/A | N/A |
| Olsson et al. | 2021 | N/A | N/A | N/A | N/A | N/A | N/A | N/A | N/A | N/A |
| Panfilov et al. | 2022 | N/A | N/A | N/A | N/A | N/A | N/A | N/A | N/A | N/A |
| Pi et al. | 2023 | N/A | N/A | N/A | N/A | N/A | N/A | N/A | N/A | N/A |
| Rahman et al. | 2022 | 0.97 | 0.964 | 0.99 | 0.96 | N/A | 0.96 | N/A | N/A | N/A |
| Rouzrokh et al. | 2023 | N/A | N/A | N/A | N/A | N/A | N/A | N/A | N/A | N/A |
| Schwartz et al. | 2020 | 0.96 | 0.93 | N/A | N/A | N/A | N/A | N/A | N/A | N/A |
| Sharma et al. | 2023 | N/A | N/A | N/A | N/A | N/A | N/A | N/A | N/A | N/A |
| Swiecicki et al. | 2021 | 0.54 | 0.81 | N/A | N/A | N/A | N/A | N/A | N/A | N/A |
| Thomas et al. | 2020 | 0.24 | 0.62 | N/A | 0.61 | N/A | 0.6 | N/A | N/A | N/A |
| Tibrewala et al. | 2020 | 0.8 | 0.65 | 0.80 | N/A | N/A | N/A | N/A | N/A | N/A |
| Tiulpin et al. | 2019 | N/A | N/A | 0.68 | N/A | N/A | N/A | N/A | N/A | N/A |
| Tiulpin et al. | 2020 | N/A | N/A | N/A | N/A | N/A | N/A | N/A | N/A | N/A |
| Tiulpin et al. | 2018 | N/A | N/A | N/A | N/A | N/A | N/A | N/A | N/A | N/A |
| Tiwari et al. | 2022 | N/A | 0.5 | N/A | 0.98 | N/A | N/A | N/A | N/A | N/A |
| Ureten et al. | 2020 | N/A | N/A | N/A | N/A | N/A | N/A | N/A | N/A | N/A |
| Vijaya et al. | 2023 | N/A | N/A | N/A | N/A | N/A | N/A | N/A | N/A | N/A |
| von Schacky et al. | 2019 | 0.58 | 0.62 | 0.58 | N/A | N/A | N/A | N/A | N/A | N/A |
| von Schacky et al. | 2020 | N/A | N/A | N/A | N/A | N/A | N/A | N/A | N/A | N/A |
| von Schacky et al. | 2020 | N/A | N/A | N/A | N/A | N/A | N/A | N/A | N/A | N/A |
| Wang et al. | 2023 | N/A | N/A | N/A | N/A | N/A | N/A | N/A | N/A | N/A |
| Wang et al. | 2022 | N/A | N/A | N/A | N/A | N/A | N/A | N/A | N/A | N/A |
| Xu et al. | 2023 | N/A | N/A | N/A | N/A | N/A | N/A | N/A | N/A | N/A |
| Yang et al. | 2022 | N/A | N/A | N/A | N/A | N/A | N/A | N/A | N/A | N/A |
| Yassine et al. | 2022 | N/A | N/A | N/A | N/A | N/A | N/A | N/A | N/A | N/A |
| Yi et al. | 2020 | N/A | N/A | N/A | N/A | N/A | N/A | N/A | N/A | N/A |
| Yi et al. | 2020 | N/A | N/A | N/A | N/A | N/A | N/A | N/A | N/A | N/A |
| Yin et al. | 2024 | N/A | N/A | N/A | N/A | N/A | N/A | N/A | N/A | N/A |
| Yoon et al. | 2023 | N/A | N/A | N/A | N/A | N/A | N/A | N/A | N/A | N/A |
| Yue et al. | 2019 | N/A | N/A | N/A | N/A | N/A | N/A | N/A | N/A | N/A |
| Yunus et al. | 2022 | N/A | N/A | N/A | N/A | N/A | N/A | N/A | N/A | N/A |

# Supplementary Material

Search terms for each of the respective databases are compiled.

## Ovid-MEDLINE

#1 (osteoarthr* or arthrit* or arthroplast* or ((knee or hip or ankle or shoulder or elbow or joint) adj3 (replacement or prosthes* or implant*)) or ((aseptic or implant or prosthetic or mechanical) adj3 loosening) or (periprosthetic adj3 (infection or fracture))).ti,ab,ot,kw,kf,hw,sy.

#2 exp Osteoarthritis/

#3 exp Arthroplasty, Replacement/

#4 exp Prosthesis-Related Infections/

#5 exp Periprosthetic Fractures/

#6 1 or 2 or 3 or 4 or 5

#7 (radiogr* or x-ray or CT or tomogr* or PET or SPECT or Triple Phase Bone Scan or MRI or "magnetic resonance" or scinti* or “bone scan”).ti,ab,ot,kw,kf,hw,sy.

#8 exp X-Rays/

#9 exp Tomography/

#10 exp Magnetic Resonance Imaging/

#11 exp Positron-Emission Tomography/

#12 exp Radionuclide Imaging/

#13 exp Radiographic Image Interpretation, Computer-Assisted/

#14 7 or 8 or 9 or 10 or 11 or 12 or 13

#15 exp Artificial Intelligence/

#16 exp Neural Networks, Computer/

#17 exp Deep Learning/

#18 (ai or “artificial intelligence” or ((machine or deep or ensemble or reinforcement) and learning) or ((deep or convolutional or neural or bayesian or recurrent or residual or artificial) adj3 network*) or perceptron or (Boltzmann adj3 machine*) or autoencoder).ti,ab,ot,kw,kf,hw,sy.

#19 15 or 16 or 17 or 18

#20 6 and 14 and 19

## Embase

#1 (osteoarthr* or arthrit* or arthroplast* or ((knee or hip or ankle or shoulder or elbow or joint) adj3 (replacement or prosthes* or implant*)) or ((aseptic or implant or prosthetic or mechanical) adj3 loosening) or (periprosthetic adj3 (infection or fracture))).ti,ab,ot,kw,sh,hw.

#2 exp osteoarthritis/

#3 exp arthroplasty/

#4 exp prosthesis infection/ or exp periprosthetic joint infection/

#5 exp periprosthetic fracture/

#6 1 or 2 or 3 or 4 or 5

#7 (radiogr* or x-ray or CT or tomogr* or PET or SPECT or MRI or "magnetic resonance" or scinti* or “bone scan”). ti,ab,ot,kw,sh,hw.

#8 exp X ray/

#9 exp x-ray computed tomography/

#10 exp nuclear magnetic resonance imaging/

#11 exp single photon emission computed tomography/

#12 exp positron emission tomography/

#13 exp bone scintiscanning/

#14 exp computer assisted diagnosis/

#15 7 or 8 or 9 or 10 or 11 or 12 or 13 or 14

#16 (ai or “artificial intelligence” or ((machine or deep or ensemble or reinforcement) and learning) or ((deep or convolutional or neural or bayesian or recurrent or residual or artificial) adj3 network*) or perceptron or (Boltzmann adj3 machine*) or autoencoder). ti,ab,ot,kw,sh,hw.

#17 exp artificial intelligence/

#18 exp deep learning/

#19 exp convolutional neural network/ or exp artificial neural network/

#20 16 or 17 or 18 or 19

#21 6 and 15 and 20

## Scopus

TITLE-ABS-KEY ( ( *arthriti* OR arthroplasty* OR ( ( knee OR hip OR ankle OR shoulder OR elbow OR joint ) PRE/3 ( replacement OR prosthes* OR implant ) ) OR ( ( aseptic OR implant OR prosthetic OR mechanical ) PRE/3 loosening ) OR ( periprosthetic PRE/3 ( infection OR fracture ) ) ) AND ( radiogr* OR x-ray OR ct OR tomogr* OR pet OR spect OR mri OR "magnetic resonance" OR scinti* OR "bone scan" ) AND ( ai OR "artificial intelligence" OR ( ( machine OR deep OR ensemble OR reinforcement ) AND learning ) OR ( ( deep OR convolutional OR neural OR bayesian OR recurrent OR residual OR artificial ) PRE/3 network* ) OR perceptron OR ( boltzmann PRE/3 machine* ) OR autoencoder ) ) AND PUBYEAR > 2011

## Web of Science

#1 TS = (*arthriti* OR arthroplasty* OR ((knee OR hip OR ankle OR shoulder OR elbow OR joint) NEAR/3 (replacement OR prosthes* OR implant)) OR ((aseptic OR implant OR prosthetic OR mechanical) NEAR/3 loosening) OR (periprosthetic NEAR/3 (infection OR fracture)))

#2 TS = (radiogr* OR x-ray OR CT OR tomogr* OR PET OR SPECT OR MRI OR “magnetic resonance” OR scinti* OR "bone scan")

#3 TS = (ai or "artificial intelligence" or ((machine or deep or ensemble or reinforcement) and learning) or ((deep or convolutional or neural or bayesian or recurrent or residual or artificial) NEAR/3 network*) or perceptron or (Boltzmann NEAR/3 machine*) or autoencoder)

#4 #1 AND #2 AND #3
